# Comparative Phytochemical Profiling and Wound Healing Potential of *Scabiosa pseudograminifolia* Hub.‐Mor. and *Scabiosa hololeuca* Bornm.: UHPLC‐HRMS/MS Analysis and Fibroblast‐Based Evaluation

**DOI:** 10.1002/fsn3.71738

**Published:** 2026-04-08

**Authors:** Kübra Öğüt, Elif Kaya Tilki, Ana B. Cerezo, Pervin Soyer, Merve Baysal, Temel Özek

**Affiliations:** ^1^ Department of Pharmacognosy Faculty of Pharmacy, Anadolu University Eskişehir Türkiye; ^2^ Department of Pharmacology Faculty of Pharmacy, Anadolu University Eskişehir Türkiye; ^3^ Departamento De Nutrición y Bromatología Toxicología y Medicina Legal. Facultad De Farmacia, Universidad De Sevilla Sevilla Spain; ^4^ Department of Pharmaceutical Microbiology Faculty of Pharmacy, Anadolu University Eskişehir Türkiye; ^5^ Department of Pharmaceutical Toxicology Faculty of Pharmacy, Anadolu University Eskişehir Türkiye

**Keywords:** fibroblast cells, *Lomelosia hololeuca*, *Lomelosia pseudograminifolia*, *UHPLC‐HRMS/MS*, wound healing

## Abstract

Species of the genus *Scabiosa* have long been used in traditional medicine for the treatment of skin disorders and inflammatory conditions; however, comparative information on their chemical composition and biological activity remains limited. In this study, the phenolic composition and functional properties of *Scabiosa pseudograminifolia* Hub.‐Mor. and *Scabiosa hololeuca* Bornm. were investigated using microwave‐assisted extraction combined with UHPLC‐HRMS/MS analysis and in vitro biological assays. Methanolic and aqueous extracts were analyzed for their phenolic constituents and evaluated in NIH/3T3 fibroblasts for intracellular reactive oxygen species (ROS) modulation, cell viability, and wound healing activity. Antimicrobial activity was additionally assessed using a broth microdilution method against selected bacterial and fungal strains. All extracts significantly reduced tert‐butyl hydroperoxide (tBHP)‐induced intracellular ROS levels, demonstrating antioxidant activity in the cellular model. Extracts of *S. pseudograminifolia* showed a pronounced wound healing effect, promoting more than 95% wound closure after 48 h at low concentrations, whereas those of *S. hololeuca* exhibited more moderate activity. The concentrations responsible for these effects were confirmed to be non‐cytotoxic. Distinct species‐ and solvent‐dependent differences in phenolic composition were observed. In *S. pseudograminifolia*, chlorogenic acid was identified as the major compound in both methanolic and aqueous extracts, while the aqueous extract also contained high levels of 4‐*O*‐caffeoylquinic acid. In contrast, *S. hololeuca* extracts contained lower amounts of chlorogenic acid and were characterized by relatively higher levels of caffeic and protocatechuic acids. Antimicrobial activity was weak to moderate, suggesting that the principal biological relevance of these extracts is associated with oxidative stress modulation and wound repair rather than direct antimicrobial effects. This study represents the first integrated phytochemical and biological evaluation of these two closely related taxa.

## Graphical abstract

Comparative phytochemical profiling of *Scabiosa pseudograminifolia* Hub.‐Mor. and *Scabiosa hololeuca* Bornm. was performed using UHPLC‐HRMS/MS, revealing phenolic‐rich extracts obtained via microwave‐assisted extraction. The extracts exhibited non‐toxic effects on NIH/3T3 cells and demonstrated wound healing, antioxidant (ROS‐modulating), and antimicrobial activities, highlighting their potential as bioactive natural sources.

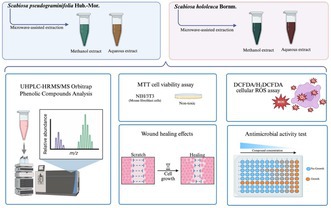

## 1 Introduction

Plants belonging to the genus *Scabiosa* (Caprifoliaceae) are widely distributed across Europe, Asia, and North Africa and have long been used in traditional medicine for the treatment of inflammatory conditions, skin disorders, and wound‐related ailments (Wen et al. 2025). In several regions, species of *Scabiosa* are commonly referred to as ‘uyuz otu’ or ‘bit otu’ in folk medicine, reflecting their traditional use in the treatment of dermatological conditions (Pinto et al. 2018; Skała and Szopa 2023). These traditional applications suggest the presence of bioactive constituents with antimicrobial, antioxidant, and tissue‐repairing properties (Albayrak et al. 2024; Kılınç et al. 2020). Notably, many *Scabiosa* species are most commonly consumed as infusions rather than applied as formulated topical preparations, indicating that their biological effects may be mediated systemically through diet‐derived bioactive constituents (Carlson et al. 2024; Kurhaluk et al. 2025; Lopes et al. 2024; Mutha et al. 2021; Wang et al. 2023).

Phytochemical investigations of various *Scabiosa* species have revealed the presence of iridoids, flavonoids, and phenolic acids–compound classes frequently associated with antioxidant, antimicrobial, and wound healing activities (Lehbili et al. 2018). Many of these compounds are recognized as bioactive phytochemicals with reported antioxidant, antimicrobial, and tissue‐protective properties, highlighting the potential nutritional and functional relevance of *Scabiosa* species (Hrichi et al. 2020; Jalloul et al. 2023; Yıldırım et al. 2025). To date, at least 256 compounds have been identified within the genus *Scabiosa*, including flavonoids (apigenin, luteolin, and quercetin derivatives), phenolic acids–particularly chlorogenic acid–triterpenoid saponins, terpenoids, fatty acids, and phytosterols (Kılınç et al. 2020; Skała and Szopa 2023; Wen et al. 2025). However, the distribution and abundance of these constituents vary considerably among species, plant parts, and extraction methods (Čulina et al. 2021; Martins Strieder et al. 2025; Sun et al. 2025). In several Iranian *Scabiosa* taxa, seed and oil fractions have been reported to contain palmitic, behenic, lignoceric, and linoleic acids as major constituents (Ebadi‐Nahari et al. 2018). Methanolic and aqueous extracts of various *Scabiosa* species have demonstrated high antioxidant capacity in DPPH, TEAC, and ORAC assays. In addition, significant α‐amylase and α‐glucosidase inhibitory activities have been reported, suggesting potential roles in glycemic modulation (Albayrak et al. 2024; Kılınç et al. 2020; Skała and Szopa 2023; Wen et al. 2025). Therefore, beyond their ethnopharmacological relevance, *Scabiosa* species have emerged as promising candidates for phenolic‐enriched functional ingredients and nutraceutical applications. Moreover, although some taxa within the genus have been studied, comprehensive and comparative phytochemical and biological evaluations remain limited for many species, (Kılınç et al. 2020; Pinto et al. 2018).

From a taxonomic perspective, the genus *Scabiosa* has undergone extensive revision, and distinctions among *Scabiosa*, *Sixalix*, and *Lomelosia* have been proposed based on morphological characteristics such as involucel structure (Carlson et al. 2009; Caputo and Del Guacchio 2011; Aksoy and Atasagun 2023). Notably, *Scabiosa hololeuca* Bornm, also referred to as *Lomelosia hololeuca* Bornm, is currently classified within the genus *Scabiosa*, reflecting its close taxonomic relationship with *Scabiosa pseudograminifolia* Hub.‐Mor. (Mayer and Ehrendorfer 1999; Caputo et al. 2004; Caputo and Del Guacchio 2011). Despite this proximity, potential differences in their chemical composition and biological activities have not been systematically explored (Skała and Szopa 2023; Wen et al. 2025).

The genus *Scabiosa* comprises numerous taxa distributed across Europe, Asia, and North Africa; however, phytochemical and functional investigations have largely concentrated on a limited number of widely distributed species (Wen et al. 2025). In contrast, *S. pseudograminifolia* and *S. hololeuca* remain comparatively underexplored despite their regional distribution and ethnobotanical relevance. While the genus has been extensively addressed from morphological and taxonomic perspectives, high‐resolution phytochemical characterization using advanced analytical techniques remains scarce for many taxa. To the best of our knowledge, this study represents the first application of microwave‐assisted extraction coupled with UHPLC‐HRMS/MS‐based phenolic profiling to *S. pseudograminifolia* and *S. hololeuca*. Moreover, no previous investigation has combined such high‐resolution metabolite profiling with functional in vitro assays to evaluate the biological relevance of these extracts.

To capture a broad spectrum of phenolic constituents with different polarities and to reflect extraction approaches relevant to traditional preparations, both methanolic and aqueous extracts were selected for investigation. Methanol is widely recognized as an efficient solvent for the recovery of phenolic acids and flavonoids, whereas aqueous extracts are particularly relevant to ethnopharmacological use and may better represent the fractions to which users are most commonly exposed (Lim et al. 2024; Sepahpour et al. 2018; Ye et al. 2015). Microwave‐assisted extraction was further employed to enhance extraction efficiency and reproducibility, enabling improved recovery of intracellular phenolics while reducing extraction time and solvent consumption (Gomez et al. 2025; Kirusnaruban et al. 2025; Oufighou et al. 2025; Tanruean et al. 2025).

Phenolic constituents commonly detected in *Scabiosa* species have been widely associated with the modulation of oxidative stress and inflammatory pathways (Rahmouni et al. 2018; Tsenguun et al. 2023). Since oxidative stress regulation and fibroblast migration represent key cellular processes involved in tissue repair, cellular antioxidant and fibroblast‐based wound healing assays provide a biologically relevant framework to evaluate the functional significance of these phytochemicals. Therefore, integrating high‐resolution phytochemical profiling with cellular antioxidant and wound healing assays allows a mechanistic assessment of the relationship between chemical composition and biological activity. In this context, antioxidant assays were used to evaluate the capacity of the extracts to modulate oxidative stress, while fibroblast‐based wound healing assays were employed to investigate their potential effects on cellular processes involved in tissue repair (Balachandran et al. 2023; Merecz‐Sadowska et al. 2021).

Given their traditional association with skin‐related uses and their predominant consumption as infusions, *Scabiosa* species represent promising candidates for phytochemical and functional investigation. Therefore, the present study integrates high‐resolution phenolic profiling with cellular antioxidant and wound healing assays to evaluate species‐dependent variability and explore the potential relationship between phenolic composition, oxidative stress modulation, and tissue‐repair processes.

## 2 Materials and Methods

### 2.1 Chemicals

Dimethyl sulfoxide (DMSO), ethanol, hexane, and methanol were purchased from Sigma‐Aldrich (St. Louis, MO, USA). Reference standards of phenolic compounds and related metabolites, including abscisic acid, 4‐hydroxybenzoic acid, 4‐*O*‐caffeoylquinic acid, caffeic acid, dihydrocaffeic acid, chlorogenic acid, *p*‐coumaric acid, gallic acid (GA), gentisic acid, *p*‐hydroxybenzoic acid, 2,4‐dihydroxybenzoic acid, protocatechuic acid, salicylic acid, sinapic acid, syringic acid, quinic acid, vanillic acid, jasmonic acid, apigenin, apigenin‐7‐*O*‐glucoside, aromadendrin, catechin, diosmetin, eriodictyol, ethyl gallate, flavanomarein, hyperoside, hesperidin, isorhamnetin, luteolin, naringin, phloretin, pinoresinol, procyanidin B1, rutin, taxifolin, quercetin, and vanillin were purchased from Sigma‐Aldrich (St. Louis, MO, USA) or Merck (Darmstadt, Germany).

### 2.2 Instruments

UHPLC‐HRMS/MS analysis was performed using a Thermo Scientific Vanquish Flex UHPLC system coupled to an Orbitrap Exploris 120 high‐resolution mass spectrometer (Thermo Fisher Scientific, Bremen, Germany) equipped with a heated electrospray ionization (HESI) source. A 12‐channel electronic pipette (Eppendorf Xplorer, Hamburg, Germany; 10–300 μL) was used for sample distribution into microplate wells. Ultrasonic‐assisted extraction was carried out using a water‐filled ultrasonic bath (Bandelin SONOREX RK 510 H, Berlin, Germany) operating at a frequency of 40 kHz with a nominal power output of 100 W.

### 2.3 Plant Material

Plant material of *S*. *pseudograminifolia* was collected in Sivas, Türkiye, at the Kangal–Gürün junction. The species was identified by Prof. Dr. Mehmet Tekin (Trakya University, Faculty of Pharmacy, Department of Pharmaceutical Botany). A voucher specimen (No. 1621) was deposited in the herbarium of Trakya University Faculty of Pharmacy.

*S. hololeuca* was collected from the western region of Eskişehir and surrounding areas of Kütahya, Türkiye. The plant material was identified by Dr. Koray Yaylacı (Anadolu University, Faculty of Pharmacy, Department of Pharmaceutical Botany). A voucher specimen was deposited in the Herbarium of Anadolu University Faculty of Pharmacy (ESSE 15843).

### 2.4 Extraction of Plant Materials

The dried aerial parts of *S. hololeuca* and *S. pseudograminifolia* were powdered and subjected to sequential extraction using methanol followed by water. Initially, the powdered plant materials were macerated with methanol under continuous shaking at room temperature for 48 h. The methanol extracts were then filtered, and the plant residues were further subjected to ultrasonic‐assisted extraction to enhance extraction efficiency. Microwave‐assisted extraction was carried out using a total solvent volume of 1200 mL under fixed‐power conditions (500 W for 3 min followed by 200 W for 30 min). The resulting supernatants were filtered through Whatman filter paper, and the methanol was removed under reduced pressure to obtain the dry methanolic extracts. The remaining plant residues were subsequently re‐extracted with water following the same procedure. Briefly, the residues were macerated with water for 48 h at room temperature under continuous shaking, followed by microwave‐assisted extraction under identical conditions (500 W for 3 min and 200 W for 30 min). The aqueous extracts were filtered and subsequently lyophilized to obtain the dry extracts. The dried extracts were stored in amber vials at 4°C until further analysis.

### 2.5 UHPLC‐HRMS/MS Orbitrap Phenolic Compounds Analysis

Phenolic compounds were analyzed using a Thermo Scientific liquid chromatography system with a binary UHPLC Dionex Ultimate 3000 RS and a Q Exactive quadrupole‐Orbitrap hybrid mass spectrometer with a HESI ionization probe. Instrument control and data acquisition were performed using Xcalibur software. Separation was done using a Waters Acquity BEH C18 column (1.7 μm particle size, 100 × 2.1 mm) at 40°C and 0.5 mL/min flow rate. A binary gradient of (A) water and (B) methanol, each containing 0.1% formic acid, was used with an elution profile of 5% B (1 min), a linear gradient to 100% B (9 min), 100% B (2 min), and 5% B (3 min). The injection volume was 5 μL. The data‐dependent acquisition approach (Top5) was used in negative mode with 70.000 and 17.500 resolutions at *m/z* 200 FWHM for full scan and product ion scan, respectively. HESI source parameters: spray voltage, −3.0 kV; S‐lens RF level, 50; capillary temperature, 320°C; sheath and auxiliary gas flow, 60 and 25 (arbitrary units); probe heater temperature, 400°C. Data processing was performed using TraceFinder software. The identification of the compound was based on the retention time, the exact masses of the pseudomolecular ion and its fragment ions (with a maximum difference of 5 ppm), and data from 87 phenolic compounds. Isotopic pattern scores of 80% were necessary (Álvarez‐Fernández et al. 2015).

### 2.6 Biological Activities

Considering their traditional use in folk medicine and their bioactive constituents, the wound healing activity of the plant extracts was investigated in healthy NIH/3T3 fibroblast cells. In addition, the effects of co‐administration with tBHP on intracellular ROS formation were evaluated in the same cell line.

Initially, the effects of the extracts and tBHP on cell viability in NIH/3T3 cells were determined using the MTT (3‐(4,5‐dimethylthiazol‐2‐yl)‐2,5‐diphenyltetrazolium bromide) assay. Based on these results, non‐cytotoxic concentrations were selected for subsequent wound healing and ROS determination experiments.

In the ROS experiments, tBHP (tert‐butyl hydroperoxide) was used as a positive control for ROS induction. Extract concentrations that exhibited positive effects on wound healing were subsequently co‐treated with the ROS‐inducing concentration of tBHP, and their potential to attenuate intracellular ROS generation was assessed.

#### 2.6.1 Cell Culture Conditions

NIH/3T3 mouse fibroblast cells were maintained in Dulbecco's Modified Eagle's Medium (DMEM) supplemented with 10% fetal bovine serum (FBS) and 1% penicillin/streptomycin. Cultures were incubated at 37°C in a humidified atmosphere with 5% CO_2_. Once the cells reached approximately 70%–80% confluence, they were either passaged into new flasks or cryopreserved for future use. Before initiating experiments, cell counts were determined using the Cedex XS system (Roche Innovatis, Germany) following trypan blue staining to ensure an appropriate number of viable cells.

#### 2.6.2 MTT Cell Viability Assay

The cytotoxic effects of the extracts of *S. pseudograminifolia* (SP_M_ and SP_W_) and *S. hololeuca* (SH_M_ and SH_W_), as well as tBHP, were evaluated using a colorimetric MTT assay in NIH/3T3 cells cultured in 96‐well plates. This assay determines cell viability based on the ability of metabolically active cells to reduce the yellow MTT compound to a purple formazan product. The intensity of the resulting color reflects the number of viable cells, with higher absorbance values indicating greater viability (Haffani et al. 2023). For the assay, cells were seeded at a density of 10 × 10^3^ cells per well and incubated for 24 h; after the incubation, the cells were treated with extract concentrations of 1, 10, 100, and 400 μg/mL and tBHP at concentrations of 1, 10, 100, and 400 μM to evaluate their effects on cell viability. Following another 24 h of incubation, 10 μL of MTT solution was added to each well. After an additional 3 h incubation, absorbance was measured at 540 nm using a Cytation 3 Cell Imaging Multi‐mode Reader (BioTek, USA). Untreated cells were used as the control group, and cell viability was expressed as a percentage relative to the control (Baghirova et al. 2023; Dikmen et al. 2017; Mosmann 1983).

#### 2.6.3 Determination of Wound Healing Effects

To evaluate the wound healing activity, an Oris Cell Migration Assay Kit (Platypus Technologies, USA) was employed. NIH/3T3 cells were seeded at a density of 5 × 10^4^ cells/well into a 96‐well plate equipped with physical barriers. After 24 h of incubation, the barriers were removed using the provided comb tool, allowing cell migration into the wound area. The cells were then incubated for an additional 24 and 48 h in 100 μL of medium containing either 1 or 10 μg/mL of SP_M_, SP_W_, SH_M_, or SH_W_, or control medium (untreated cells). Images of the wound area were captured at 0, 24, and 48 h using a Leica DM300 light microscope at 4× magnification. Wound diameters were measured using ImageJ software, and the changes were expressed as a percentage relative to the initial (0 h) wound size (Baghirova et al. 2023).

#### 2.6.4 DCFDA/H_2_DCFDA Cellular ROS Assay

To determine whether the SP_M_, SP_W_, SH_M_, and SH_W_ extracts at wound healing effective concentrations of 1 and 10 μg/mL reduce reactive oxygen species (ROS) formation when co‐administered with tBHP, intracellular ROS measurement was performed. For this purpose, the DCFDA/H_2_DCFDA Cellular ROS Assay Kit (Abcam PLC, Cambridge, MA) was used in accordance with the manufacturer's instructions. Cells were seeded in 96‐well plates at a density of 2.5 × 10^4^ cells/100 μL and incubated for 24 h. After incubation, the medium was removed, and 100 μL of DCFDA (20 μM) solution was added to each well and incubated for 45 min. Following this period, the DCFDA solution was discarded, and the wells were washed with 1X buffer. Subsequently, 100 μM tBHP, a ROS inducer, was applied together with the extracts (1 and 10 μg/mL). Cells treated with 0.05% dimethyl sulfoxide (DMSO) served as the vehicle control. Fluorescence intensity was measured after 4 h using a fluorescence plate reader (excitation: 485 nm/emission: 535 nm) (Baysal et al. 2024).

#### 2.6.5 Antimicrobial Activity Test

##### 2.6.5.1 Determination of Minimum Inhibitory Concentration (MIC)

The test was carried out using *Bacillus subtilis* NRRL B478, *Staphylococcus aureus* ATCC 6538, *Pseudomonas aeruginosa* ATCC 27853, *Escherichia coli* ATCC 3521, *Candida albicans* ATCC 90028, and *Candida krusei* ATCC 62582. Fresh 24 h cultures of the test microorganisms incubated at 37°C were adjusted to a defined cell density using the McFarland standard. The microbial cell densities in physiological saline solution (FTS) were adjusted to 1.5 × 10^8^ CFU/mL according to McFarland 0.5, and then diluted to 2.5–5 × 10^5^ CFU/mL for bacterial cultures (CLSI, 2007) and 0.5–2.5 × 10^3^ CFU/mL for yeast cultures. Bacteria were suspended in MHB medium, while *Candida* species were suspended in SDB medium (CLSI, 2002; 2007; 2012). Stock solutions of all extracts were prepared at 60000 μg/mL (60 mg/mL) by dissolving the methanol and aqueous extracts of *S. hololeuca* (SH_M_, SH_W_) and *S. pseudograminifolia* (SP_M_, SP_W_) in 10% DMSO–methanol. All extracts were dissolved in 10% DMSO‐methanol. Two‐fold serial dilutions were subsequently performed across wells 1–12 of sterile 96‐well microplates, yielding a final concentration range of 15,000–3.6621 μg/mL for the antimicrobial assays. Then, 100 μL of microbial cultures were added to each well, and the plates were incubated at 37°C for 24 h. Standard antimicrobial agents were included as positive controls. Erythromycin (5–0.001 μg/mL) was used against Gram‐positive bacteria, ciprofloxacin (4–0.0009 μg/mL) against Gram‐negative bacteria, and amphotericin B (4–0.0009 μg/mL) against yeast strains. Then, 100 μL of microbial cultures were added to each well, and the plates were incubated at 37°C for 24 h. Positive controls included different concentrations of standard antibiotics: erythromycin for Gram‐positive bacteria, ciprofloxacin for Gram‐negative bacteria, and amphotericin B for yeasts. After incubation, 20 μL of 0.01% resazurin solution was added to the wells, followed by an additional 3 h of incubation at 37°C. A color change from blue to pink indicated microbial growth, whereas wells that remained blue‐purple were considered growth‐inhibited.

### 2.7 Statistical Analyses

Cell viability and wound healing data were analyzed and visualized using GraphPad Prism 7.0 software (GraphPad Software, USA). Statistical analysis was performed using one‐way ANOVA followed by Tukey's post hoc test. Results are presented as mean ± standard deviation (SD) from two independent experiments (*n* = 6 for the cell viability assay; *n* = 4 for the wound healing assay). Statistical significance was defined as *p < 0.05, **p < 0.01, ***p < 0.001, and ****p < 0.0001 compared to the control group; values with *p* > 0.05 were considered not significant (ns).

Statistical analyses of the DCFDA/H_2_DCFDA cellular ROS assay results were performed using GraphPad Prism 9.0 software (GraphPad Software, USA). The distribution of the data was assessed using the Shapiro–Wilk test, and variance homogeneity was examined using the Brown–Forsythe test. For datasets meeting normality and homogeneity assumptions, one‐way ANOVA was applied, followed by Dunnett's post hoc test for multiple comparisons. Data are expressed as mean ± standard deviation (SD), and statistical significance was defined as *p* < 0.05.

## 3 Results and Discussion

### 3.1 Yields Obtained

Amounts of extracts and yields of methanolic and aqueous extracts are summarized in Table 1. The extraction method integrated traditional maceration with microwave‐assisted extraction to improve extraction efficiency. Variations in extraction yields were noted between the two species and the solvents employed.

**TABLE 1:** Extraction yields and extract amounts of methanolic and aqueous extracts from *S. hololeuca* and *S. pseudograminifolia*.

	*S. pseudograminifolia*		*S. hololeuca*	
	Amounts of extracts, g	Yield %^a^	Amounts of extracts, g	Yield %^a^
Methanol extract	6.45	16.12	7.74	19.34
Aqueous extract	7.49	18.72	8.12	20.30

^a^ Extraction yield (%) was calculated based on the dry weight of the plant material.

The extraction yields of *S. hololeuca* and *S. pseudograminifolia* were contingent upon the solvent employed, with aqueous extracts producing higher yields than methanol extracts for both species. The elevated extraction yields noted for these *Scabiosa* species (Akar 2021; Albayrak et al. 2024; Jalloul et al. 2023) can be ascribed to the utilization of microwave‐assisted extraction, which improves solvent penetration and mass transfer efficacy. The observed differences between the two species further illustrate species‐specific matrix features that affect extraction efficiency (Bagade and Patil 2021; Gil‐Martín et al. 2022).

### 3.2 UHPLC‐HRMS/MS Orbitrap Phenolic Compounds Analysis

In this study, both qualitative and quantitative analyses of the phenolic compounds were conducted on the methanol and aqueous extracts of *S. hololeuca and S. pseudograminifolia* aerial parts. Qualitative identification of the compounds was performed based on retention times, exact mass measurements, and MS/MS fragmentation patterns. Following qualitative characterization, quantitative determination of the identified phenolic compounds was carried out using calibration curves constructed from a 12‐point serial dilution of a standard mixture prepared from commercially available reference compounds. Compound concentrations were calculated accordingly. The combined qualitative and quantitative data obtained from the methanolic and aqueous extracts of *S. pseudograminifolia* are presented in Data S1 and S2, whereas the corresponding results for the methanolic and aqueous extracts of *S. hololeuca* are provided in Data S3 and S4. The quantitative results of the identified compounds are summarized in Table 2.

**TABLE 2:** Quantitative phenolic composition of methanolic and aqueous extracts of *S. pseudograminifolia* and *S. hololeuca* determined by UHPLC‐HRMS/MS.

Compound	SP_M_ (μ g_compound_/g_plant_)	SP_W_ (μ g_compound_/g_plant_)	SH_M_ (μ g_compound_/g_plant_)	SH_W_ (μ g_compound_/g_plant_)
4‐Hydroxybenzoic acid	299.18 ± 11.02	229.57 ± 3.09	123.46 ± 9.40	148.23 ± 0.46
4‐*O*‐Caffeoylquinic acid	N.d.	11519.15 ± 703.70	2891.07 ± 117.13	1906.38 ± 34.83
Caffeic acid	405.70 ± 13.58	217.49 ± 0.82	832.43 ± 0.92	6384.86 ± 22.80
Chlorogenic acid	19839.62 ± 421.20	25949.47 ± 36.45	14150.41 ± 26.12	1307.87 ± 29.77
Gentisic acid	N.d.	N.d.	58.26 ± 0.96	245.09 ± 18.24
*p*‐Coumaric acid	320.46 ± 1.51	57.86 ± 0.04	97.16 ± 2.24	747.55 ± 11.26
Protocatechuic acid	462.90 ± 5.21	161.47 ± 6.80	357.05 ± 4.30	1640.79 ± 23.92
Apigenin‐7‐*O*‐glucoside	723.79 ± 5.32	235.12 ± 1.38	421.14 ± 4.38	N.d.
Diosmetin	N.d.	N.d.	73.61 ± 0.63	84.89 ± 0.94
Luteolin	40.41 ± 14.68	199.49 ± 4.43	147.68 ± 1.07	150.99 ± 4.47
Naringin	N.d.	N.d.	7.29 ± 0.01	N.d.
Vanillin	207.03 ± 7.93	75.75 ± 0.93	100.25 ± 14.26	92.15 ± 2.09

*Note:* Values are expressed as mean ± SD (*n* = 2).

Abbreviation: N.d., not detected.

The UHPLC‐HRMS/MS Orbitrap study demonstrated different species‐ and solvent‐dependent variations in the phenolic composition of *S. pseudograminifolia* and *S. hololeuca*. Chlorogenic acid has been found as the major phenolic compound in the methanolic extract of *S. pseudograminifolia*, quantified at 19839.62 ± 421.20 μg_compound_/g_plant_, followed by apigenin‐7‐*O*‐glucoside at 723.79 ± 5.32 μg_compound_/g_plant_. Conversely, the aqueous extract of *S. pseudograminifolia* was primarily composed of chlorogenic acid (25949.47 ± 36.45 μg_compound_/g_plant_) and 4‐*O*‐caffeoylquinic acid (11519.15 ± 703.70 μg_compound_/g_plant_). Chlorogenic acid was identified as the major compound in the methanolic extract of *S. hololeuca* (14150.41 ± 26.12 μg_compound_/g_plant),_ alongside 4‐*O*‐caffeoylquinic acid (2891.07 ± 117.13 μg_compound_/g_plant_). The aqueous extract displayed a more varied phenolic composition, with caffeic acid identified as the major component (6384.86 ± 22.80 μg_compound_/g_plant_), succeeded by 4‐*O*‐caffeoylquinic acid (1906.38 ± 34.83 μg compound/g_plant_) and protocatechuic acid (1640.79 ± 23.92 μg_compound_/g_plant_). Prior research on *Scabiosa* species has indicated substantially lower or inconsistent levels of major phenolic compounds in comparison to the findings of the current study (Jalloul et al. 2022; Öğüt et al. 2025a; Öğüt, et al. 2025b; Rahmouni, et al. 2018; Skała and Szopa 2023; Yıldırım et al. 2025). In the methanolic flower extract of *S. columbaria* subsp. *columbaria* var. *columbaria*, 4‐hydroxybenzoic acid (42.21 μg/g) and caffeic acid (26.28 μg/g) were recognized as major compounds, but the methanolic leaf extract was primarily characterized by 4‐hydroxybenzoic acid at 19.54 μg/g (Akar 2021). In *S. stellata* , whole‐plant extracts were identified to include isoorientin (66.31 ± 0.30 mg/g_extract_) and 4‐*O*‐caffeoylquinic acid (26.41 ± 0.30 mg/g_extract_) as the major compounds (Rahmouni, et al. 2018). Extracts of *S. atropurpurea* , derived using chloroform, dichloromethane, ethyl acetate, and ethanol, contained chlorogenic acid in concentrations ranging from 6.08 to 657.78 μg/g and cynaroside (luteolin‐7‐*O*‐glucoside) from 39.79 to 741.60 μg/g (Hrichi et al. 2020). In *S. maritima* , leaf‐derived acetate extracts exhibited significant concentrations of catechin hydrate (1951.86–6748.97 mg/100 g_extract_), in addition to substantial quantities of luteolin‐7‐*O*‐glucoside and kaempferol‐3‐*O*‐rutinoside (Jalloul et al. 2022). Prior phytochemical investigations of *S. hololeuca* and *S. pseudograminifolia* have mainly used traditional maceration techniques combined with RP‐HPLC analysis, documenting specific phenolic acids at comparatively low concentrations, at the μg/g_extract_ level. The investigations revealed the presence of simple phenolic acids, including *o*‐coumaric, caffeic, syringic, ferulic, and *p*‐coumaric acids in *S. hololeuca*, and a comparable phenolic acid profile in *S. pseudograminifolia*, with biological activity mostly linked to total phenolic content (Öğüt et al. 2025b; Öğüt, et al. 2025a). Conversely, the current study utilized microwave‐assisted extraction combined with UHPLC‐HRMS/MS orbitrap analysis, resulting in enhanced extraction efficiency and increased analytical sensitivity. The significantly elevated concentrations and more extensive phenolic profiles noted in this study, especially for chlorogenic acid and caffeoylquinic acid derivatives, are most likely ascribed to variations in extraction methodology and analytical precision rather than to inconsistencies in plant chemistry. Accordingly, the quantitative and qualitative disparities between the present findings and previous publications are predominantly attributed to differences in extraction methodologies and analytical resolution, rather than fundamental alterations in species chemistry. The findings illustrate the importance of sophisticated extraction methodologies and high‐resolution mass spectrometry in achieving thorough and dependable phenolic profiling within the genus *Scabiosa*.

Several of the phenolic compounds identified in the present study have previously been associated with antioxidant and tissue‐repair related biological activities (Melguizo‐Rodríguez, Illescas‐Montes, et al. 2021). Chlorogenic acid and caffeic acid derivatives are widely reported to exhibit strong antioxidant and anti‐inflammatory properties, which are important processes in the wound healing cascade (Nguyen et al. 2024; S. Wang et al. 2024; Wang et al. 2026). Likewise, protocatechuic acid has been shown to contribute to cellular protection against oxidative stress (Lee and Lee 2022). Flavonoids such as apigenin‐7‐*O*‐glucoside, luteolin, and diosmetin are also known to modulate inflammatory mediators, regulate reactive oxygen species, and support fibroblast migration and tissue regeneration (Caporali et al. 2022; Che et al. 2020; Melguizo‐Rodríguez, Luna‐Bertos, et al. 2021). Therefore, the presence of these phenolic constituents suggests that *Scabiosa* extracts may contribute to biologically relevant properties related to oxidative stress modulation and tissue repair.

### 3.3 Biological Activities

#### 3.3.1 Effects of the Extracts and tBHP on NIH/3 T3 Cell Viability

**FIGURE 1:**
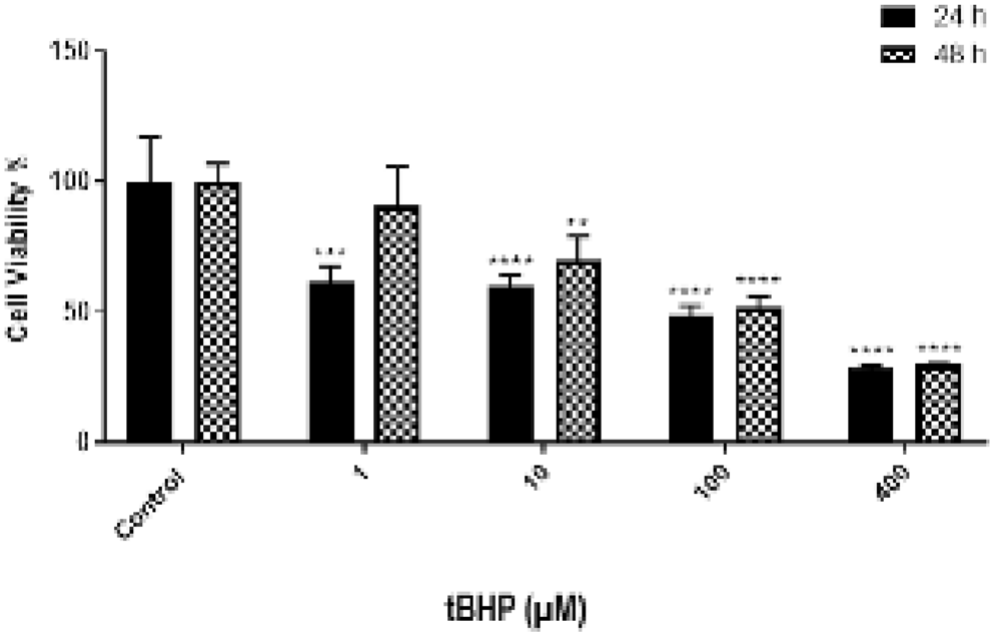
Cell viability assay results for varying concentrations (1, 10, 100, and 400 μM) of tBHP on NIH/3T3 cells. Viability was assessed after 24 and 48 h of treatment, and the results are presented as mean ± SD of two independent experiments (*n* = 6 for each treatment group). Statistical significance was defined as *p < 0.05, **p < 0.01, ***p < 0.001, and ****p < 0.0001; ns indicates no significant difference.

The cytotoxic effects of tBHP, SP_M_, SP_W_, SH_M_, and SH_W_ on NIH/3T3 cells were assessed through an MTT assay. Following 24‐h exposure to tBHP at concentrations of 400, 100, 10, and 1 μM, cell viability was reduced to 28.4%, 49%, 60.46%, and 61.71%, respectively. After 48 h, the viability percentages at the same concentrations were 29.84%, 51.64%, 69.82%, and 90.82%, respectively (Figure 1). The IC_50_ values of tBHP in NIH/3T3 cells were calculated as 126.43 μM at 24 h and 103.61 μM at 48 h. Based on these results, 100 μM tBHP was chosen as the working concentration for the following assays.

**FIGURE 2:**
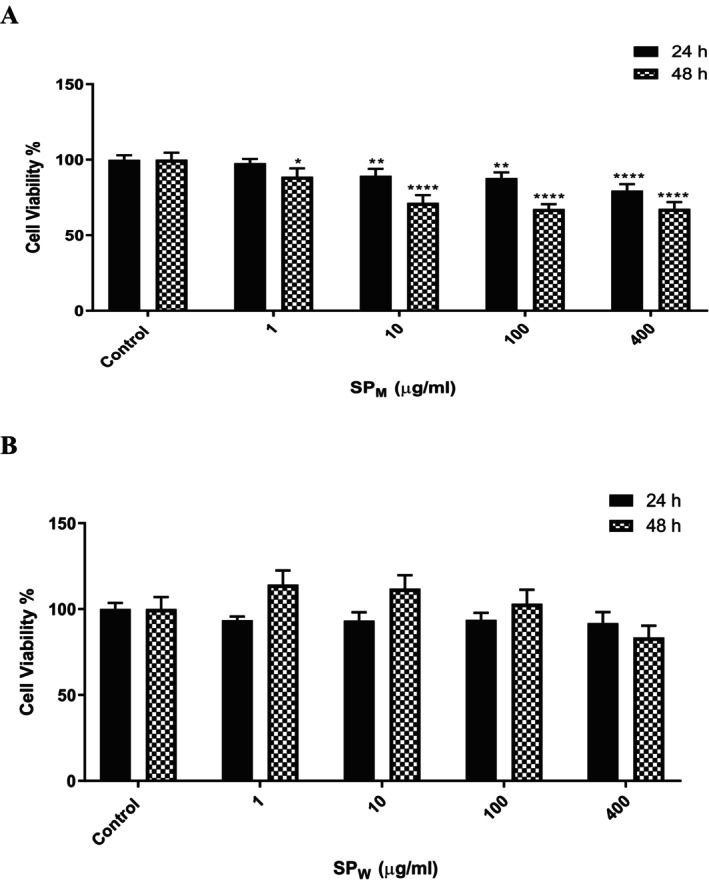
Cell viability assay results for varying concentrations (1, 10, 100, and 400 μg/mL) of SP_M_ (A) and SP_W_ (B) on NIH/3T3 cells. Viability was assessed after 24 and 48 h of treatment. Data are expressed as mean ± SD from two independent experiments (*n* = 6 per treatment group). Statistical significance was defined as *p < 0.05, **p < 0.01, ***p < 0.001, and ****p < 0.0001; ns indicates no significant difference.

Treatment of NIH/3T3 cells with SP_W_ at concentrations of 400, 100, 10, and 1 μg/mL for 24 h resulted in cell viability values of 40.03%, 46.95%, 67.34%, and 85.00%, respectively. Under the same conditions, SP_M_ treatment resulted in viability values of 91.78%, 93.40%, 93.28%, and 93.51%, respectively (Figure 2). After 48 h of exposure, cell viability values for SP_W_ at the same concentrations were 83.44%, 103.62%, 111.89%, and 114.25%, respectively, whereas SP_M_ produced viability values of 67.43%, 67.40%, 71.39%, and 88.71%, respectively (Figure 2).

**FIGURE 3:**
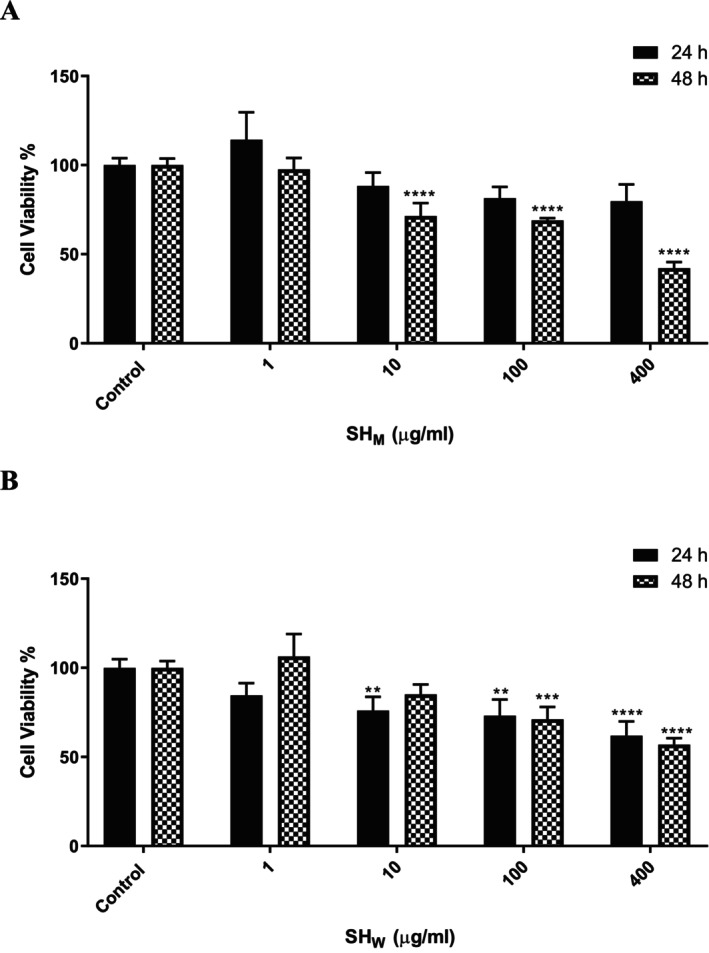
Cell viability assay results for varying concentrations (1, 10, 100, and 400 μg/mL) of SH_M_ (A) and SH_W_ (B) on NIH/3T3 cells. Viability was assessed after 24 and 48 h of treatment. Data are expressed as mean ± SD from two independent experiments (*n* = 6 per treatment group). Statistical significance was defined as **p < 0.01, ***p < 0.001, and ****p < 0.0001; ns indicates no significant difference.

After 24 h of treatment with SH_W_, and SH_M_ at concentrations of 400, 100, 10, and 1 μg/mL, cell viability was reduced to 23.21%, 30.07%, 83.41%, and 96.36%; 61.9%, 70.67%, 76.01%, and 84.54%; and 79.79%, 81.45%, 88.29%, and 114.25%, respectively. After 48 h, the viability percentages at the same concentrations were 15.74%, 21.95%, 59.67%, and 96.87%; 56.94%, 74.13%, 85.13%, and 106.36%; and 42.13%, 69.03%, 71.38%, and 97.55%, respectively (Figure 3).

At lower concentrations (1–10 μg/mL), the extracts did not exhibit cytotoxic effects and in some cases slightly increased cell viability, suggesting a potential proliferative or cytoprotective effect in NIH/3T3 fibroblasts.

#### 3.3.2 Effects of the Extracts on Wound Healing

The wound closure effects of SP‐derived extracts (SP_M_ and SP_W_) were evaluated at 10 and 1 μg/mL concentrations over 24 and 48 h. At 24 h, SP_M_ treatment reduced wound area to 79.52% (10 μg/mL) and 81.97% (1 μg/mL), while SP_W_ resulted in 80.06% and 76.31% wound areas, respectively, indicating moderate closure.

**FIGURE 4:**
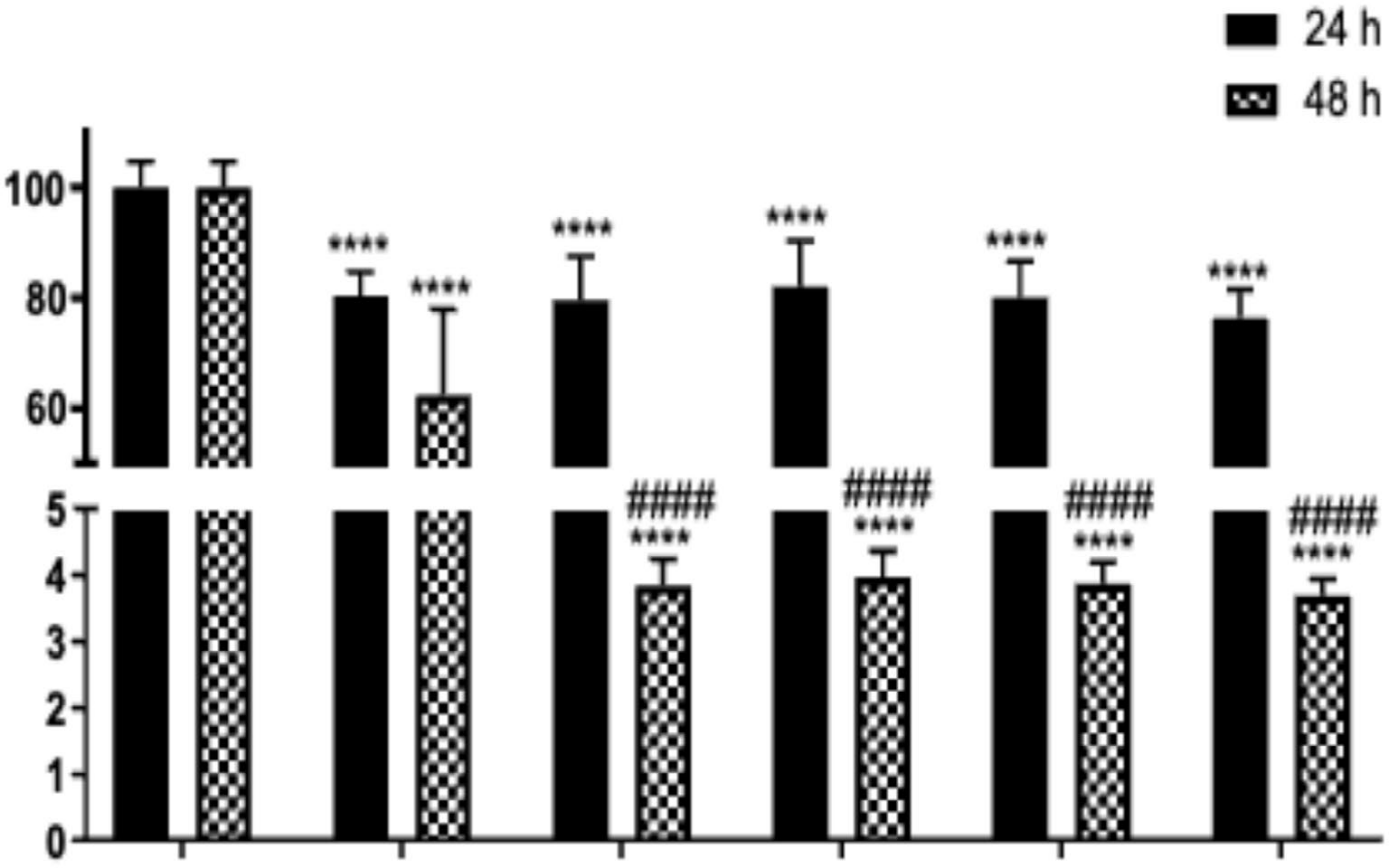
Wound closure percentages at 24 and 48 h following treatment with SP extracts at 10 and 1 μg/mL. Data were normalized to the 0 h time point and are expressed as mean ± SD from two independent experiments (*n* = 4 per treatment group). Statistical significance was defined as ****p < 0.0001 vs 0 h and ****p < 0.0001 vs control; ns indicates no significant difference.

By 48 h, both SP_M_ and SP_W_ exhibited remarkable wound healing activity. The wound area decreased to 3.85% (SP_M_ 10 μg/mL), 3.97% (SP_M_ 1 μg/mL), 3.87% (SP_W_ 10 μg/mL), and 3.69% (SP_W_ 1 μg/mL), reflecting over 95% closure in all treatment groups. These findings suggest that SP‐derived methanol and aqueous extracts, even at low concentrations, are highly effective in promoting wound healing in NIH/3T3 cells (Figure 4).

**FIGURE 5:**
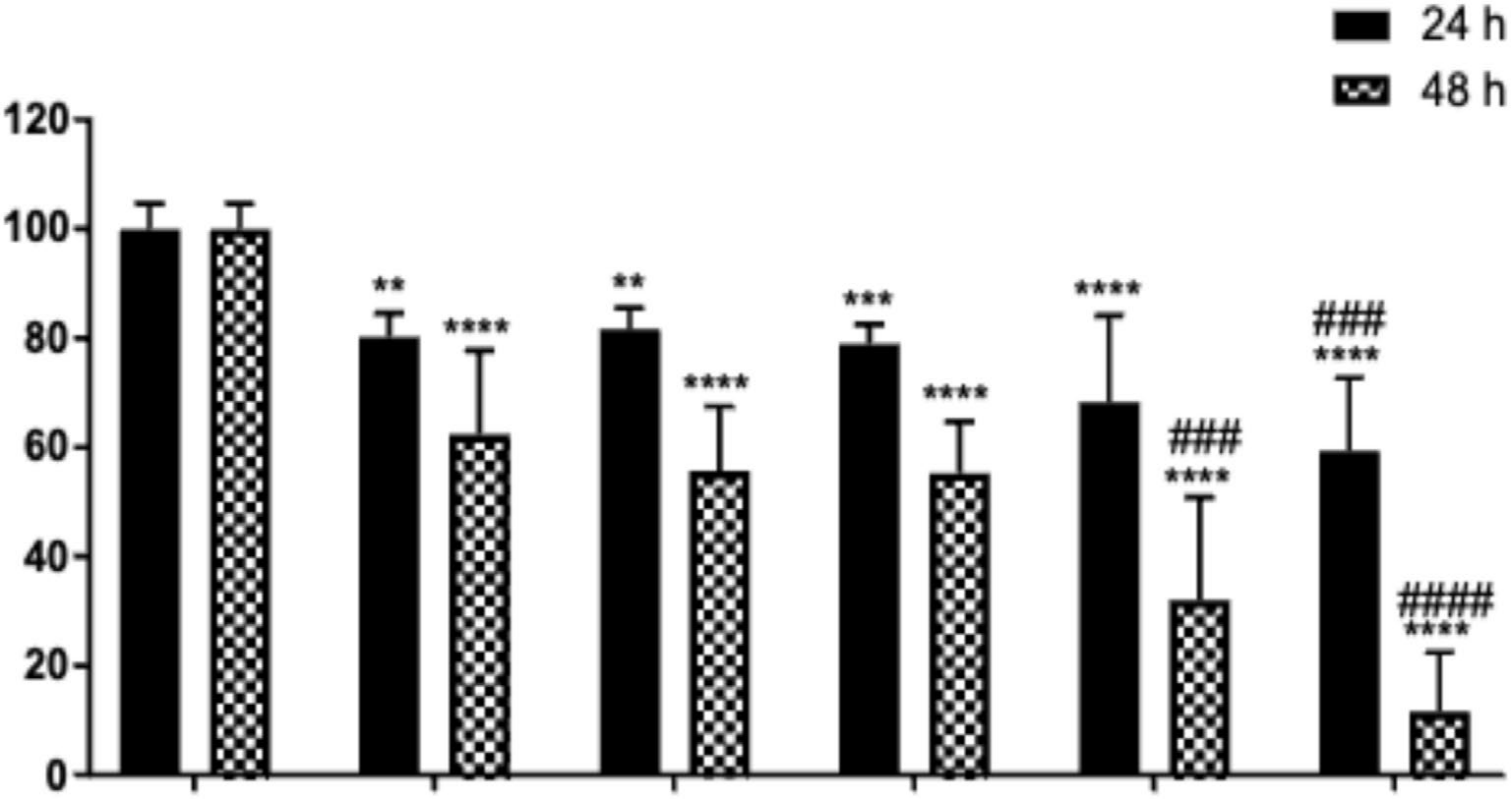
Wound closure percentages at 24 and 48 h following treatment with SH extracts at concentrations of 10 and 1 μg/mL. Data were normalized to the 0 h time point and are expressed as mean ± SD from two independent experiments (*n* = 4 per treatment group). Statistical significance was defined as **p < 0.01, ***p < 0.001, and ****p < 0.0001 vs 0 h; ^###^ *p* < 0.001 and ^####^ *p* < 0.0001 vs. the control group; ns indicates no significant difference.

Wound healing activity of SH‐derived extracts (SH_M_ and SH_W_) was also assessed under the same conditions. At 24 h, SH_M_‐treated cells showed relatively high residual wound areas: 81.77% (10 μg/mL) and 79.04% (1 μg/mL), indicating modest closure. SH_W_ exhibited slightly greater wound closure at this time point, reducing the wound area to 68.25% (10 μg/mL) and 59.33% (1 μg/mL). However, at 48 h, SH‐derived extracts were markedly less effective compared to SP extracts. SH_M_‐treated groups showed wound areas of 55.66% (10 μg/mL) and 55.33% (1 μg/mL), while SH_W_‐treated cells retained 32.13% (10 μg/mL) and 11.63% (1 μg/mL) of the initial wound. These data indicate that SH extracts exhibit limited wound healing capacity, especially at higher concentrations (Figure 5).

Although a direct migration assay was not conducted, the remarkable wound closure observed in the SP_M_‐treated group–despite only moderate levels of cell viability–suggests that the methanolic extract of SP may promote wound closure partly through enhanced fibroblast migration. This hypothesis is supported by the disparity between the extent of wound closure and cell viability at 48 h: while SP_M_ maintained approximately 67% viability, it achieved over 96% wound closure. In contrast, SH_M_ exhibited similar cell viability but significantly less wound healing activity, indicating a possible difference in migratory response. Further studies incorporating dedicated migration assays would help validate this observation. The current findings demonstrate that extracts from *S. pseudograminifolia* (SP_M_ and SP_W_) demonstrated stronger biological effects compared to those from *S. hololeuca* (SH_M_ and SH_W_) in the NIH/3T3 fibroblast model. SP‐derived extracts demonstrated stronger wound closure effects compared with SH extracts, achieving over 95% closure after 48 h even at low concentrations (1–10 μg/mL). In contrast, SH‐derived extracts had comparatively limited effects under the same conditions. To our knowledge, this study provides the first experimental evidence of wound healing and intracellular ROS‐modulating properties of *Scabiosa* species in a fibroblast‐based in vitro model. These differences indicate species‐dependent variation in the biological potential within the genus *Scabiosa*. This means that it is vital to pick the proper species when undertaking biological testing on *Scabiosa* taxa. These findings designate *S. pseudograminifolia* as a notable candidate for further mechanistic and in vivo, investigations.

#### 3.3.3 DCFDA/H_2_DCFDA Cellular ROS Assay

**FIGURE 6:**
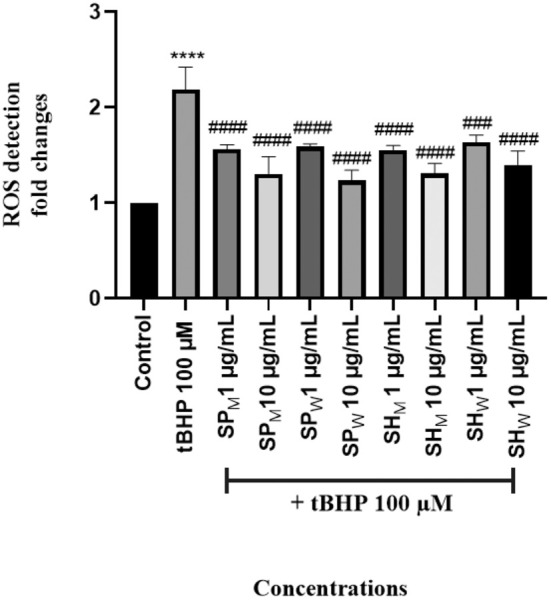
Protective effects of SP_M_, SP_W_, SH_M_, and SH_W_ extracts against *t*BHP‐induced ROS formation in NIH/3T3 cells. Data were calculated as fold changes relative to the control group and are expressed as mean ± SD from three independent experiments. *p <* 0.05 vs. control; ^#^ *p* < 0.05 vs. tBHP. Statistical significance was indicated according to the GraphPad Prism style *p* > 0.1234 (ns); *p* ≤ 0.0332 (* or #); *p* ≤ 0.0021 (* or ##); *p* ≤ 0.0002 (* or ###); *p* < 0.0001 (* or ####).

The experimental results were evaluated by calculating fold changes relative to the control group. Treatment with 100 μM tBHP induced ROS formation, leading to a 2.2‐fold increase in NIH/3T3 cells. However, when SH_M_, SH_W_, SP_M_, and SP_W_ extracts were applied at concentrations of 1 and 10 μg/mL simultaneously with tBHP, the fold changes decreased to 1.2–1.6 relative to the control. These results indicate that co‐treatment with the extracts attenuated ROS generation. Moreover, this reduction was found to be more pronounced with increasing extract concentrations (Figure 6).

In our study, SP_M_, SP_W_, SH_M_, and SH_W_ extracts were found to reduce ROS levels at the same concentrations (1 and 10 μg/mL) at which they exhibited wound healing activity in the same cell model. This observation is consistent with the presence of phenolic compounds identified in the UHPLC‐HRMS/MS analysis, many of which are known to possess antioxidant and cytoprotective properties. This finding suggests that the wound healing effects of these extracts may be associated not only with enhanced cell proliferation but also with attenuation of oxidative stress. As highlighted in the literature, ROS play an essential role in wound healing by contributing to antibacterial defense, platelet activation, and angiogenesis. However, excessive ROS production induces oxidative stress, thereby disrupting the balance between pro‐oxidants and antioxidants, which is associated with delayed wound healing, particularly in chronic wounds and metabolic disorders. The role of antioxidants in mitigating oxidative stress and accelerating wound repair is increasingly recognized. Indeed, antioxidants such as vitamins E and C, curcumin, ferulic acid, and resveratrol have been shown to promote wound closure, reduce oxidative damage, and enhance the activity of antioxidant enzymes (Baysal et al. 2024; Ukaegbu et al. 2025).

Our findings demonstrate that the investigated extracts possess both antioxidant properties and wound‐healing potential, in line with the existing literature. However, more comprehensive and detailed studies are required to fully elucidate the underlying mechanisms of these effects. Although direct comparative studies examining ROS modulation in fibroblast models using *Scabiosa* species are currently lacking, the present study offers the initial evidence that extracts derived from *S. hololeuca* and *S. pseudograminifolia* can mitigate tBHP‐induced intracellular ROS production in NIH/3T3 fibroblast cells. This response aligns with prior findings concerning phenolic‐rich plant extracts in analogous cellular oxidative stress scenarios. In the context of wound healing, careful modulation of ROS is essential, since high oxidative stress is recognized to hinder fibroblast function and postpone tissue restoration. Consequently, the simultaneous decrease in ROS levels and enhancement of wound healing activity seen in this investigation indicate a supplementary antioxidant role in the biological effects of the examined extracts.

#### 3.3.4 Antimicrobial Activity Test

The antimicrobial activities of the methanolic and aqueous extracts of *S. pseudograminifolia* (SP_M_ and SP_W_) and *S. hololeuca* (SH_M_ and SH_W_) were evaluated using the broth microdilution method. The tested concentration ranges were 15.000–0.003 μg/mL for SP_M_, SH_M_, and SH_W_, and 7500–1.83 μg/mL for SP_W_. Minimum inhibitory concentrations (MICs) were determined against a panel of Gram‐positive and Gram‐negative bacteria, as well as yeast strains. The results are presented in Table 3.

**TABLE 3:** Minimum inhibitory concentrations (MIC, μg/mL) of *S. pseudograminifolia* and *S. hololeuca* extracts against selected bacterial and fungal strains.

Extracts	*B. subtilis*	*S. aureus*	*P. aeruginosa*	*E. coli*	*C. albicans*	*C. krusei*
SP_M_	7500	7500	7500	7500	7500	7500
SP_W_	7500	7500	7500	7500	≥ 3750	≥ 3750
SH_M_	7500	3750	7500	1850	7500	7500
SH_W_	≥ 15,000	≥ 15,000	≥ 15,000	≥ 15,000	≥ 15,000	≥ 15,000
Erythromycin	1.25	1.25	—	—	—	—
Ciprofloxacin	—	—	1	0.0156	—	—
Amphotericin B	—	—	—	—	0.5	1

The antimicrobial activity of the extracts showed clear species‐ and solvent‐dependent differences. The methanolic extract of *S. hololeuca* (SH_M_) exhibited moderate antibacterial activity against selected strains, whereas its aqueous extract (SH_W_) showed no detectable antibacterial effect within the tested concentration range (MIC ≥ 15,000 μg/mL), indicating limited antimicrobial potential of water‐soluble constituents from this species under the applied conditions. In contrast, both methanolic (SP_M_) and aqueous (SP_W_) extracts of *S. pseudograminifolia* displayed consistent antibacterial activity against all tested bacterial strains, with MIC values of 7500 μg/mL, although their antifungal activity remained limited (MIC ≥ 3750 μg/mL). These findings indicate that *S. pseudograminifolia* exhibits a broader and more consistent antibacterial profile than *S. hololeuca* under the present experimental conditions. To place these results in a broader context, antimicrobial activities reported for other *Scabiosa* species are summarized below. A study on *S. arenaria* reported notable antimicrobial activity for butanol fractions obtained from flowers, fruits, and stems/leaves against *E. coli* , *P. aeruginosa* , and *C. albicans* . The strongest effect was observed against *E. coli* , with a MIC as low as 0.0195 mg/mL (Besbes Hlila et al. 2016). Extracts of *S. olivieri* have been reported to exhibit antimicrobial activity against both *E. coli* and *S. aureus* , with MIC values of 9.37 and 4.68 mg/mL, respectively, indicating moderate antibacterial efficacy (Yıldırım et al. 2025). Crude extracts and newly isolated bis‐iridoid compounds from *S. stellata* have demonstrated antimicrobial activity against *E. faecalis* and *S. epidermidis* , with minimum inhibitory concentration (MIC) values ranging from 31.2 to 62.5 μg/mL for the bis‐iridoid constituents, indicating potent activity at the microgram‐per‐milliliter level (Lehbili et al. 2018). Extracts of *S. hymettia* have been reported to exhibit the highest antimicrobial activity among the tested samples against a panel of six bacterial and three fungal strains, indicating broad‐spectrum and comparatively strong antimicrobial potential within the genus (Christopoulou et al. 2008). Stem extracts of *S. atropurpurea* have been reported to exhibit considerable antibacterial activity against several bacterial strains, while showing comparatively weaker effects against fungal species, suggesting a preferential antibacterial profile (Hrichi et al. 2020). Various fractions obtained from the fruits of *S. rotata* have been reported to show no detectable antibacterial activity against *E. coli* , *P. aeruginosa* , *E. faecalis* , or *S. aureus* , indicating a lack of antibacterial efficacy for this plant part and species under the tested conditions (Yıldırım et al. 2025). Taken together, these reports indicate that antimicrobial potency within the genus varies widely depending on species, plant part, extraction solvent, and the degree of compound purification. In this context, the present study provides the first experimental evaluation of the antimicrobial activity of *S. pseudograminifolia* and *S. hololeuca* using standardized crude extract assays.

## 4 Conclusion

The present study employed microwave‐assisted extraction combined with UHPLC‐HRMS/MS analysis to enable efficient recovery and comprehensive characterization of phenolic compounds from *S. pseudograminifolia* and *S. hololeuca*. The phytochemical profiling revealed that hydroxycinnamic acid derivatives, particularly chlorogenic acid and caffeoylquinic acid isomers, were among the major constituents of the extracts.

Biological evaluations demonstrated that the extracts–especially those obtained from *S. pseudograminifolia*–significantly reduced intracellular reactive oxygen species (ROS) levels, preserved NIH/3T3 fibroblast viability, and promoted wound closure at low concentrations. Considering the well‐documented antioxidant and cytoprotective properties of phenolic acids and flavonoids, these findings suggest that the observed wound healing effects may be associated with modulation of oxidative stress and stimulation of fibroblast‐related repair processes.

Despite the close taxonomic relationship between the two taxa, notable differences were observed in both phenolic composition and biological activity. These results highlight the importance of taxon‐level differentiation in phytochemical and pharmacological investigations and emphasize the relevance of species selection when evaluating medicinal plants for therapeutic applications.

Overall, this study represents the first comprehensive phytochemical and biological investigation of *S. pseudograminifolia* and *S. hololeuca*, providing a valuable basis for future studies aimed at isolating the active compounds and elucidating their mechanisms of action in wound healing and oxidative stress‐related processes.

## Author Contributions

**Kübra Öğüt:** conceptualization, data curation, formal analysis, funding acquisition, investigation, methodology, resources, software, validation, visualization, writing – original draft, writing – review and editing. **Elif Kaya Tilki:** conceptualization, data curation, formal analysis, investigation, methodology, resources, software, validation, visualization, writing – original draft. **Ana B. Cerezo:** conceptualization, data curation, formal analysis, investigation, methodology, project administration, software, validation, visualization, writing – original draft, writing – review and editing. **Pervin Soyer:** conceptualization, data curation, formal analysis, investigation, methodology, resources, software, validation, visualization, writing – original draft. **Merve Baysal:** conceptualization, data curation, formal analysis, investigation, methodology, resources, software, validation, visualization, writing – original draft. **Temel Özek:** conceptualization, data curation, formal analysis, investigation, project administration, validation, visualization, writing – original draft, writing – review and editing.

## Funding

The authors have nothing to report.

## Conflicts of Interest

The authors declare no conflicts of interest.

## Supporting information


**Data S1:** Quantified Phenolic Compounds in the Methanol Extracts of the Aerial Parts of *S. pseudograminifolia*.

**Data S2:** Quantified Phenolic Compounds in the Aqueous Extracts of the Aerial Parts of *S. pseudograminifolia*.

**Data S3:** Quantified Phenolic Compounds in the Methanol Extracts of the Aerial Parts of *S. hololeuca*.

**Data S4:** Quantified Phenolic Compounds in the Aqueous Extracts of the Aerial Parts of *S. hololeuca*.

## Data Availability

The data that support the findings of this study are available from the corresponding author upon reasonable request.

## References

[fsn371738-bib-0001] Akar, Z. 2021. “Chemical Compositions by Using LC–MS/MS and GC–MS and Antioxidant Activities of Methanolic Extracts From Leaf and Flower Parts of *Scabiosa columbaria* subsp. *columbaria* var. *columbaria* L.” Saudi Journal of Biological Sciences 28, no. 11: 6639–6644. 10.1016/j.sjbs.2021.07.039. 34764779 PMC8568819

[fsn371738-bib-0002] Aksoy, A. , and B. Atasagun . 2023. “Pollen Morphology of *Scabiosa* L. and *Lomelosia* Raf.(Caprifoliaceae) Taxa in Türkiye.” Turkish Journal of Botany 47, no. 6: 567–585. 10.55730/1300-008X.2785.

[fsn371738-bib-0003] Albayrak, S. , A. Aksoy , M. A. Yilmaz , and E. Beyzi . 2024. “Investigation of Phytochemical, Antioxidant and Antidiabetic Potentials of *Scabiosa* L.(Caprifoliaceae) Species With Chemometric Methods.” Chemistry and Biodiversity 21, no. 2: e202301652. 10.1002/cbdv.202301652. 38240171

[fsn371738-bib-0004] Álvarez‐Fernández, M. A. , A. B. Cerezo , A. M. Canete‐Rodriguez , A. M. Troncoso , and M. C. García‐Parrilla . 2015. “Composition of Nonanthocyanin Polyphenols in Alcoholic‐Fermented Strawberry Products Using LC–MS (QTRAP), high‐Resolution MS (UHPLC‐Orbitrap‐MS), LC‐DAD, and Antioxidant Activity.” Journal of Agricultural and Food Chemistry 63, no. 7: 2041–2051. 10.1021/jf506076n. 25598511

[fsn371738-bib-0005] Bagade, S. B. , and M. Patil . 2021. “Recent Advances in Microwave Assisted Extraction of Bioactive Compounds From Complex Herbal Samples: A Review.” Critical Reviews in Analytical Chemistry 51, no. 2: 138–149. 10.1080/10408347.2019.1686966. 31729248

[fsn371738-bib-0006] Baghirova, L. , E. Kaya Tilki , and A. A. Ozturk . 2023. “Evaluation of Cell Proliferation and Wound Healing Effects of Vitamin A Palmitate‐Loaded PLGA/Chitosan‐Coated PLGA Nanoparticles: Preparation, Characterization, Release, and Release Kinetics.” ACS Omega 8, no. 2: 2658–2668. 10.1021/acsomega.2c07232. 36687101 PMC9851036

[fsn371738-bib-0007] Balachandran, A. , S. N. Siyumbwa , G. R. A. Froemming , et al. 2023. “in vitro Antioxidant and Fibroblast Migration Activities of Fractions Eluded From Dichloromethane Leaf Extract of *Marantodes pumilum* .” Life 13, no. 6: 1409. 10.3390/life13061409. 37374190 PMC10304334

[fsn371738-bib-0008] Baysal, M. , A. B. Karaduman , B. Korkut Çelikateş , Ö. Atlı‐Eklioğlu , and S. Ilgın . 2024. “Assessment of the Toxicity of Different Antiretroviral Drugs and Their Combinations on Sertoli and Leydig Cells.” Drug and Chemical Toxicology 47, no. 6: 1100–1108. 10.1080/01480545.2024.2336506. 38647040

[fsn371738-bib-0009] Besbes Hlila, M. , H. Mosbah , K. Majouli , et al. 2016. “Antimicrobial Activity of *Scabiosa arenaria* Forssk. Extracts and Pure Compounds Using Bioguided Fractionation.” Chemistry and Biodiversity 13, no. 10: 1262–1272. 10.1002/cbdv.201600028. 27448449

[fsn371738-bib-0010] Caporali, S. , A. De Stefano , C. Calabrese , et al. 2022. “Anti‐Inflammatory and Active Biological Properties of the Plant‐Derived Bioactive Compounds Luteolin and Luteolin 7‐Glucoside.” Nutrients 14, no. 6: 1155. 10.3390/nu14061155. 35334812 PMC8949538

[fsn371738-bib-0011] Caputo, P. , S. Cozzolino , and A. Moretti . 2004. “Molecular Phylogenetics of Dipsacaceae Reveals Parallel Trends in Seed Dispersal Syndromes.” Plant Systematics and Evolution 246, no. 3–4: 163–175. 10.1007/s00606-004-0154-y.

[fsn371738-bib-0012] Caputo, P. , and E. Del Guacchio . 2011. “Transfer of Four Species of *Scabiosa* to *Lomelosia* (Dipsacaceae).” Novon: A Journal for Botanical Nomenclature 21, no. 4: 402–404. 10.3417/2009135.

[fsn371738-bib-0013] Carlson, D. A. , C. True , and C. G. Wilson . 2024. “Oxidative Stress and Food as Medicine.” Frontiers in Nutrition 11: 1394632. 10.3389/fnut.2024.1394632. 39262430 PMC11387802

[fsn371738-bib-0014] Carlson, S. E. , V. Mayer , and M. J. Donoghue . 2009. “Phylogenetic Relationships, Taxonomy, and Morphological Evolution in Dipsacaceae (Dipsacales) Inferred by DNA Sequence Data.” Taxon 58, no. 4: 1075–1091. 10.1002/tax.584003.

[fsn371738-bib-0015] Che, D. N. , B. O. Cho , J. Kim , J. Y. Shin , H. J. Kang , and S. I. Jang . 2020. “Luteolin and Apigenin Attenuate LPS‐Induced Astrocyte Activation and Cytokine Production by Targeting MAPK, STAT3, and NF‐κB Signaling Pathways.” Inflammation 43, no. 5: 1716–1728. 10.1007/s10753-020-01245-6. 32462548

[fsn371738-bib-0016] Christopoulou, C. , K. Graikou , and I. Chinou . 2008. “Chemosystematic Value of Chemical Constituents From *Scabiosa hymettia* (Dipsacaceae).” Chemistry and Biodiversity 5, no. 2: 318–323. 10.1002/cbdv.200890029. 18293445

[fsn371738-bib-0017] Čulina, P. , D. Cvitković , D. Pfeifer , et al. 2021. “Phenolic Profile and Antioxidant Capacity of Selected Medicinal and Aromatic Plants: Diversity Upon Plant Species and Extraction Technique.” PRO 9, no. 12: 2207. 10.3390/pr9122207.

[fsn371738-bib-0018] Dikmen, M. , Z. Canturk , E. K. Tilki , and S. Engur . 2017. “Evaluation of Antiangiogenic and Antimetastatic Effects of *Penicillium chrysogenum* Secondary Metabolites.” Indian Journal of Pharmaceutical Sciences 79, no. 1: 49–57.

[fsn371738-bib-0019] Ebadi‐Nahari, M. , P. Farnia , S. Nikzat , and S. Mollaei . 2018. “A Chemotaxonomic Evaluation of Some *Scabiosa* L. Species in Iran.” Biochemical Systematics and Ecology 81: 33–36. 10.1016/j.bse.2018.09.003.

[fsn371738-bib-0020] Gil‐Martín, E. , T. Forbes‐Hernández , A. Romero , D. Cianciosi , F. Giampieri , and M. Battino . 2022. “Influence of the Extraction Method on the Recovery of Bioactive Phenolic Compounds From Food Industry By‐Products.” Food Chemistry 378: 131918. 10.1016/j.foodchem.2021.131918. 35085901

[fsn371738-bib-0021] Gomez, P. , C. Reyes , and J. G. Figueroa . 2025. “Microwave‐Assisted Extraction of Phenolic Compounds From *Cocoa pod* Husk: Process Optimization and Impact of Drying Temperature on Bioactive Recovery.” Molecules 30, no. 17: 3497. 10.3390/molecules30173497. 40942025 PMC12429909

[fsn371738-bib-0022] Haffani, Y. Z. , K. Louati , E. K. Tilki , et al. 2023. “Anti‐Tumoral Activity of *Allium roseum* Compounds on Breast Cancer Cells MCF7 and MDA‐MB231.” Advances in Traditional Medicine 24, no. 1: 323–333. 10.1007/s13596-023-00699-x.

[fsn371738-bib-0023] Hrichi, S. , R. Chaabane‐Banaoues , S. Bayar , et al. 2020. “Botanical and Genetic Identification Followed by Investigation of Chemical Composition and Biological Activities on the *Scabiosa atropurpurea* L. Stem From Tunisian Flora.” Molecules 25, no. 21: 5032. 10.3390/molecules25215032. 33138334 PMC7684468

[fsn371738-bib-0024] Jalloul, A. b. , H. Chaar , M. S. Tounsi , and M. Abderrabba . 2022. “Variations in Phenolic Composition and Antioxidant Activities of *Scabiosa maritima* ( *Scabiosa atropurpurea* *subsp. maritima* L.) Crude Extracts and Fractions According to Growth Stage and Plant Part.” South African Journal of Botany 146: 703–714. 10.1016/j.sajb.2021.12.004.

[fsn371738-bib-0025] Jalloul, A. B. , S. Garzoli , H. Chaar , C. el Jribi , and M. Abderrabba . 2023. “Seasonal Effect on Bioactive Compounds Recovery Using Aqueous Extraction, Antioxidant Activities, and Volatile Profiles of Different Parts of *Scabiosa maritima* L (= *Scabiosa atropurpurea subsp. maritima* (L.) Arcang.).” South African Journal of Botany 152: 63–79. 10.1016/j.sajb.2022.11.031.

[fsn371738-bib-0027] Kirusnaruban, K. , N. Gasparre , R. Nandasiri , M. N. A. Eskin , and C. M. Rosell . 2025. “Green Extraction of Wheat Phenolic Acids Using Microwave‐Assisted Extraction.” Journal of Food Science 90, no. 5: e70225. 10.1111/1750-3841.70225. 40331797 PMC12057545

[fsn371738-bib-0026] Kılınç, H. , M. Masullo , G. D'Urso , T. Karayildirim , O. Alankus , and S. Piacente . 2020. “Phytochemical Investigation of *Scabiosa sicula* Guided by a Preliminary HPLC‐ESIMSn Profiling.” Phytochemistry 174: 112350. 10.1016/j.phytochem.2020.112350. 32208198

[fsn371738-bib-0028] Kurhaluk, N. , L. Buyun , R. Kołodziejska , P. Kamiński , and H. Tkaczenko . 2025. “Effect of Phenolic Compounds and Terpenes on the Flavour and Functionality of Plant‐Based Foods.” Nutrients 17, no. 21: 3319. 10.3390/nu17213319. 41228391 PMC12609125

[fsn371738-bib-0029] Lee, W.‐J. , and S.‐H. Lee . 2022. “Protocatechuic Acid Protects Hepatocytes Against Hydrogen Peroxide‐Induced Oxidative Stress.” Current Research in Food Science 5: 222–227. 10.1016/j.crfs.2022.01.006. 35106486 PMC8789513

[fsn371738-bib-0030] Lehbili, M. , A. A. Magid , J. Hubert , et al. 2018. “Two New Bis‐Iridoids Isolated From *Scabiosa stellata* and Their Antibacterial, Antioxidant, Anti‐Tyrosinase and Cytotoxic Activities.” Fitoterapia 125: 41–48. 10.1016/j.fitote.2017.12.018. 29273413

[fsn371738-bib-0031] Lim, J. , K. Kim , D. Y. Kwon , J. K. Kim , R. Sathasivam , and S. U. Park . 2024. “Effects of Different Solvents on the Extraction of Phenolic and Flavonoid Compounds, and Antioxidant Activities, in *Scutellaria baicalensis* Hairy Roots.” Horticulturae 10, no. 2: 160. 10.3390/horticulturae10020160.

[fsn371738-bib-0032] Lopes, F. B. , M. M. Sarandy , R. D. Novaes , G. Valacchi , and R. V. Gonçalves . 2024. “OxInflammatory Responses in the Wound Healing Process: A Systematic Review.” Antioxidants 13, no. 7: 823. 10.3390/antiox13070823. 39061892 PMC11274091

[fsn371738-bib-0033] Martins Strieder, M. , I. Lopes de Oliveira , F. Sanchez Bragagnolo , et al. 2025. “Consistency of Phenolic Compounds in Plant Residues Parts: A Review of Primary Sources, Key Compounds, and Extraction Trends.” Journal of Agricultural and Food Chemistry 73, no. 19: 11515–11534. 10.1021/acs.jafc.5c01868. 40300049 PMC12082697

[fsn371738-bib-0034] Mayer, V. , and F. Ehrendorfer . 1999. “Fruit Differentiation, Palynology, and Systematics in the *Scabiosa* Group of Genera and Pseudoscabiosa (Dipsacaceae).” Plant Systematics and Evolution 216, no. 1: 135–166.

[fsn371738-bib-0035] Melguizo‐Rodríguez, L. , E. de Luna‐Bertos , J. Ramos‐Torrecillas , R. Illescas‐Montesa , V. J. Costela‐Ruiz , and O. García‐Martínez . 2021. “Potential Effects of Phenolic Compounds That Can Be Found in Olive Oil on Wound Healing.” Food 10, no. 7: 1642. 10.3390/foods10071642. PMC8307686 34359512

[fsn371738-bib-0036] Melguizo‐Rodríguez, L. , R. Illescas‐Montes , V. J. Costela‐Ruiz , et al. 2021. “Antimicrobial Properties of Olive Oil Phenolic Compounds and Their Regenerative Capacity Towards Fibroblast Cells.” Journal of Tissue Viability 30, no. 3: 372–378. 10.1016/j.jtv.2021.03.003. 33810929

[fsn371738-bib-0037] Merecz‐Sadowska, A. , P. Sitarek , E. Kucharska , et al. 2021. “Antioxidant Properties of Plant‐Derived Phenolic Compounds and Their Effect on Skin Fibroblast Cells.” Antioxidants 10, no. 5: 726. 10.3390/antiox10050726. 34063059 PMC8147979

[fsn371738-bib-0038] Mosmann, T. 1983. “Rapid Colorimetric Assay for Cellular Growth and Survival: Application to Proliferation and Cytotoxicity Assays.” Journal of Immunological Methods 65, no. 1–2: 55–63. 6606682 10.1016/0022-1759(83)90303-4

[fsn371738-bib-0039] Mutha, R. E. , A. U. Tatiya , and S. J. Surana . 2021. “Flavonoids as Natural Phenolic Compounds and Their Role in Therapeutics: An Overview.” Future Journal of Pharmaceutical Sciences 7, no. 1: 25. 10.1186/s43094-020-00161-8. 33495733 PMC7816146

[fsn371738-bib-0040] Nguyen, V. , E. G. Taine , D. Meng , T. Cui , and W. Tan . 2024. “Chlorogenic Acid: A Systematic Review on the Biological Functions, Mechanistic Actions, and Therapeutic Potentials.” Nutrients 16, no. 7: 924. 10.3390/nu16070924. 38612964 PMC11013850

[fsn371738-bib-0042] Öğüt, K. , G. Özek , N. Öztürk , M. Tekin , and T. Özek . 2025b. “Comprehensive Investigation of Phytochemical Constituents and Biological Activities of *Scabiosa pseudograminifolia* Hub.‐Mor.” Turkish Journal of Pharmaceutical Sciences 22, no. 2: 104. 10.4274/tjps.galenos.2025.12247. 40366211 PMC12080293

[fsn371738-bib-0041] Öğüt, K. , G. Özek , N. Öztürk , Ö. K. Yaylacı , and T. Özek . 2025a. “Chemical Composition, α‐Amylase Inhibition, and Antioxidant Activities of *Scabiosa hololeuca* Bornm.” Biochemical Systematics and Ecology 122: 105028. 10.1016/j.bse.2025.105028.

[fsn371738-bib-0043] Oufighou, A. , F. Brahmi , S. Achat , et al. 2025. “Optimization of Microwave‐Assisted Extraction of Phenolic Compounds From *Opuntia ficus‐indica* Cladodes.” PRO 13, no. 3: 724. 10.3390/pr13030724.

[fsn371738-bib-0044] Pinto, D. C. G. A. , N. Rahmouni , N. Beghidja , and A. M. S. Silva . 2018. “ *Scabiosa* Genus: A Rich Source of Bioactive Metabolites.” Medicine 5, no. 4: 110. 10.3390/medicines5040110. PMC6313729 30304864

[fsn371738-bib-0045] Rahmouni, N. , D. C. G. A. Pinto , N. Beghidja , S. Benayache , and A. M. S. Silva . 2018. “ *Scabiosa stellata* L. Phenolic Content Clarifies Its Antioxidant Activity.” Molecules 23, no. 6: 1285. 10.3390/molecules23061285. 29861483 PMC6100036

[fsn371738-bib-0046] Rahmouni, N. , D. C. G. A. Pinto , S. A. O. Santos , N. Beghidja , and A. M. S. Silva . 2018. “Lipophilic Composition of *Scabiosa stellata* L.: An Underexploited Plant From Batna (Algeria).” Chemical Papers 72, no. 3: 753–762. 10.1007/s11696-017-0308-3.

[fsn371738-bib-0047] Sepahpour, S. , J. Selamat , M. Y. Abdul Manap , A. Khatib , and A. F. Abdull Razis . 2018. “Comparative Analysis of Chemical Composition, Antioxidant Activity and Quantitative Characterization of Some Phenolic Compounds in Selected Herbs and Spices in Different Solvent Extraction Systems.” Molecules 23, no. 2: 402. 10.3390/molecules23020402. 29438306 PMC6017614

[fsn371738-bib-0048] Skała, E. , and A. Szopa . 2023. “ *Dipsacus* and *Scabiosa* Species–The Source of Specialized Metabolites With High Biological Relevance: A Review.” Molecules 28, no. 9: 3754. 10.3390/molecules28093754. 37175164 PMC10180103

[fsn371738-bib-0049] Sun, S. , Y. Yu , Y. Jo , et al. 2025. “Impact of Extraction Techniques on Phytochemical Composition and Bioactivity of Natural Product Mixtures.” Frontiers in Pharmacology 16: 1615338. 10.3389/fphar.2025.1615338. 40808686 PMC12343529

[fsn371738-bib-0050] Tanruean, K. , S. Luangkamin , T. Srisurat , W. Bunmusik , and P. Suttiarporn . 2025. “Optimization of Microwave‐Assisted Extraction Process for Production of Polyphenol‐Rich Crude Extract From *Cinnamomum iners* Leaves.” Applied Sciences (2076–3417) 15, no. 3: 1265. 10.3390/app15031265.

[fsn371738-bib-0051] Tsenguun, T. , A. Altanchimeg , G. Soyolmaa , et al. 2023. “Extract of *Scabiosa comosa* Exhibits an Anti‐Inflammatory Effect on Carrageenan and Lipopolysaccharide‐Induced Acute Inflammation in Rats.” International Journal of Pharmacology 19, no. 2: 157–165. 10.3923/ijp.2023.157.165.

[fsn371738-bib-0052] Ukaegbu, K. , E. Allen , and K. K. H. Svoboda . 2025. “Reactive Oxygen Species and Antioxidants in Wound Healing: Mechanisms and Therapeutic Potential.” International Wound Journal 22, no. 5: e70330. 10.1111/iwj.70330. 40288766 PMC12034374

[fsn371738-bib-0053] Wang, G. , F. Yang , W. Zhou , N. Xiao , M. Luo , and Z. Tang . 2023. “The Initiation of Oxidative Stress and Therapeutic Strategies in Wound Healing.” Biomedicine and Pharmacotherapy 157: 114004. 10.1016/j.biopha.2022.114004. 36375308

[fsn371738-bib-0054] Wang, S. , Y. Liu , X. Wang , et al. 2024. “Modulating Macrophage Phenotype for Accelerated Wound Healing With Chlorogenic Acid‐Loaded Nanocomposite Hydrogel.” Journal of Controlled Release 369: 420–443. 10.1016/j.jconrel.2024.03.054. 38575075

[fsn371738-bib-0055] Wang, Y. Q. , L. Y. Jia , S. H. Shen , et al. 2026. “Anti‐Inflammatory and Tissue Regeneration Effects of a Chlorogenic Acid/Hyaluronic Acid Hydrogel on Methicillin‐Resistant *Staphylococcus aureus* ‐Infected Diabetic Wounds.” Biomaterials Science 14, no. 1: 240–255. 10.1039/d5bm01236h. 41222509

[fsn371738-bib-0056] Wen, R. , W. Feng , N. Wang , et al. 2025. “Research Advances in the Genus *Scabiosa*: A Comprehensive Review of Botany, Traditional Uses, Phytochemistry, and Pharmacology.” Chemistry and Biodiversity 22, no. 9: e202500229. 10.1002/cbdv.202500229. 40198120

[fsn371738-bib-0057] Ye, F. , Q. Liang , H. Li , and G. Zhao . 2015. “Solvent Effects on Phenolic Content, Composition, and Antioxidant Activity of Extracts From Florets of Sunflower ( *Helianthus annuus* L.).” Industrial Crops and Products 76: 574–581. 10.1016/j.indcrop.2015.07.063.

[fsn371738-bib-0058] Yıldırım, A. , İ. K. Celep , A. Gül , Ö. S. Eter , and E. Bağcı . 2025. “Unveiling the Bioactive Potential of *Scabiosa rotata* M. Bieb Fruits: A Landmark Study on Cytotoxic, Antioxidant, and Antibacterial Activities Through Liquid Chromatography‐Mass Spectrometry Profiling and Molecular Docking.” International Journal of Food Science and Technology 60, no. 1: vvaf098. 10.1093/ijfood/vvaf098.

