# Supplementary material for: Comparative Phytochemical Profiling and Wound Healing Potential of Scabiosa pseudograminifolia Hub.‐Mor. and Scabiosa hololeuca Bornm.: UHPLC‐HRMS/MS Analysis and Fibroblast‐Based Evaluation
Source: Food Sci Nutr. 2026 Apr 8;14(4):e71738. doi: 10.1002/fsn3.71738 (PMC13058435; doi:10.1002/fsn3.71738)
Supplement: Supplementary file 1 — Data S1: Quantified Phenolic Compounds in the Methanol Extracts of the Aerial Parts of S. pseudograminifolia. [file FSN3-14-e71738-s002.docx]

**Supplementary Material 1.** Quantified Phenolic Compounds in the Methanol Extracts of the Aerial Parts of S. pseudograminifolia

| **Identification** | **t_R_ (min)** | **Molecular formula** | **Exact mass**  **(M −H )^−^** | ***m/z* (Expected)** | ***m/z* (Apex)** | **Δmass (ppm)** | **MS/MS fragments** | **Mg_compound_/g_plant_**  **_(medium_ _±std)_** |
| --- | --- | --- | --- | --- | --- | --- | --- | --- |
| 4-Hydroxybenzoic acid | 3.38 | C_7_H_6_O_3_ | 137.0244 | 137.02442 | 137.02432 | 0.71148 | 65.03979; 93.03467; 137.024568 | 299.18±11.02 |
| 4-O-Caffeoylquinic acid | 3.85 | C_16_H_18_O_9_ | 353.0878 | 353.08781 | 353.08768 | 0.37667 | 93.03467; 135.04524; 173.04582; 179.03545; 191.05643 | N.d. |
| Abscisic acid | 6.57 | C_15_H_20_O_4_ | 263.1288 | 263.12888 | 263.12878 | 0.36416 | 122.03728; 153.09229; 204.11578; 204.1161; 219.13924 | N.d. |
| Caffeic acid | 4.02 | C_9_H_8_O_4_ | 179.0350 | 179.03498 | 179.0349 | 0.12352 | 89.03979; 107.05037; 134.03752; 135.04535; 179.03525 | 405.70±13.58 |
| Chlorogenic acid | 3.86 | C_16_H_18_O_9_ | 353.08781 | 353.08781 | 353.08771 | 0.29024 | 59.01394; 85.02962; 93.03467; 127.0402; 191.05643 | 19839.62±421.20 |
| Gallic Acid | 1.11 | C_7_H_6_O_5_ | 169.01425 | 169.01425 | 169.01424 | 0,08017 | 69.03467; 79.01913; 81.0347; 97.0296; 125.02451 | N.d. |
| *p*-Coumaric acid | 4.82 | C_9_H_8_O_3_ | 163.0395 | 163.04007 | 163.04002 | 0.37693 | 65.03967; 91.05541; 93.03464; 104.02694; 119.05029 | 320.46±1.51 |
| Protocatechuic acid | 2.2 | C_7_H_6_O_4_ | 153.0193 | 153.01933 | 153.01924 | 0.47973 | 65.00341; 81.03467; 91.01905; 108.02183; 109.0259 | 462.90±5.21 |
| Quinic acid | 0.53 | C_7_H_12_O_6_ | 191.05611 | 191.05611 | 191.05606 | 0.25756 | 85.0296; 93.03465; 109.02959; 127.04026; 173.04597 | N.d. |
| Apigenin-7-O-Glc | 6.01 | C_21_H_20_O_10_ | 432.10555 | 431.09837 | 431.09811 | 0.59380 | 63.02412; 107.01402; 117.03471; 211.04037; 268.03806 | 723.79±5.32 |
| Apigenin | 7.32 | C_15_H_10_O_5_ | 270.05282 | 269.04555 | 269.04544 | 0.40635 | 65.0034; 107.01382; 117.03468; 149.0244; 151.00378 | N.d. |
| Diosmetin | 7.39 | C_16_H_12_O_6_ | 299.0561 | 299.05611 | 299.05585 | 0.87887 | 63.02399; 65.00335; 107.01402; 151.00386; 284.03287 | N.d. |
| Hyperoside | 5.64 | C_21_H_20_O_12_ | 464.0955 | 463.0882 | 463.08829 | 0.18863 | 227.03545; 243.02998; 255.03024; 271.02518;300.02783 | N.d. |
| Luteolin | 6.9 | C_15_H_10_O_6_ | 286.0404 | 285.04046 | 285.04034 | 0.40612 | 65.00337; 107.01404; 151.00389; 175.04042 | 40.41±14.68 |
| Vanilin | 4.49 | C_8_H_8_O_3_ | 151.04006 | 151.04007 | 151.03993 | 0.91200 | 108.02187; 151.04048 | 207.03±7.93 |

**t_R:_** retention time; *m/z* (Expected): theoretical mass-to-charge ratio calculated from the molecular formula; *m/z* (Apex): experimentally observed value at the chromatographic peak apex; Δmass (ppm): mass error between theoretical and observed *m/z* values; N.d.: Not detected.
