# Supplementary material for: Comparative Phytochemical Profiling and Wound Healing Potential of Scabiosa pseudograminifolia Hub.‐Mor. and Scabiosa hololeuca Bornm.: UHPLC‐HRMS/MS Analysis and Fibroblast‐Based Evaluation
Source: Food Sci Nutr. 2026 Apr 8;14(4):e71738. doi: 10.1002/fsn3.71738 (PMC13058435; doi:10.1002/fsn3.71738)
Supplement: Supplementary file 2 — Data S2: Quantified Phenolic Compounds in the Aqueous Extracts of the Aerial Parts of S. pseudograminifolia. [file FSN3-14-e71738-s004.docx]

**Supplementary Material 2.** Quantified Phenolic Compounds in the Aqueous Extracts of the Aerial Parts of S. pseudograminifolia

| **Identification** | **t_R_ (min)** | **Molecular formula** | **Exact mass**  **(M −H )^−^** | ***m/z* (Expected)** | ***m/z* (Apex)** | **Δmass (ppm)** | **MS/MS fragments** | **Mg_compound_/g_plant_**  **_(medium_ _±std)_** |
| --- | --- | --- | --- | --- | --- | --- | --- | --- |
| 4-Hydroxybenzoic acid | 3.38 | C_7_H_6_O_3_ | 137.0244 | 137.02442 | 137.02431 | 0.82284 | 93.03465; 137.02457 | 229.57±3.09 |
| 4-O-Caffeoylquinic acid | 4.04 | C_16_H_18_O_9_ | 353.0878 | 353.08781 | 353.08786 | 0.14191 | 93.03465; 135.04532; 173.04573; 179.03535; 191.05646 | 11519.15±703.70 |
| Caffeic acid | 4,05 | C_9_H_8_O_4_ | 179.0350 | 179.03498 | 179.0349 | 0.46443 | 89.03975; 107.05026; 134.03754; 135.04538; 179.03525 | 217.49±0.82 |
| Chlorogenic acid | 3.86 | C_16_H_18_O_9_ | 353.08781 | 353.08781 | 353.08768 | 0.37667 | 59.01392; 85.02961; 93.03465; 127.04021; 191.05646 | 25949.47±36.45 |
| *p-*Coumaric acid | 4.81 | C_9_H_8_O_3_ | 163.0395 | 163.04007 | 163.04002 | 0.28334 | 65.03958; 91.05543; 93.03464; 119.05034 | 57.86±0.04 |
| Protocatechuic acid | 2.2 | C_7_H_6_O_4_ | 153.0193 | 153.01933 | 153.01924 | 0.67917 | 65.00336; 81.03463; 91.01899; 108.02184; 109.0296 | 161.47±6.80 |
| Quinic acid | 0.52 | C_7_H_12_O_6_ | 191.05611 | 191.05611 | 191.05597 | 0,73676 | 85.02957; 93.03462; 109.02956; 127.04016; 173.04572 | N.d. |
| Apigenin-7-O-Glc | 6.01 | C_21_H_20_O_10_ | 432.105554 | 431.09837 | 431.09802 | 0.80617 | 63.02409; 107.01395; 117.03468; 211.04053; 268.03806 | 235.12±1.38 |
| Hyperoside | 5.64 | C_21_H_20_O_12_ | 464.0955 | 463.0882 | 463.08847 | 0.58403 | 227.03534; 243.03011; 255.03024; 271.02518; 300.02783 | N.d. |
| Luteolin | 6.9 | C_15_H_10_O_6_ | 286.0404 | 285.04046 | 285.04031 | 0.51319 | 65.00341; 107.01388; 133.02963; 151.00343; 175.04015 | 199.49±4.43 |
| Naringin | 5.82 | C_27_H_32_O_14_ | 579.1719 | 579.17193 | 579.17169 | 0.41111 | 65.00337; 107.01395; 119.05031; 151.00381; 271.06158 | 75.75±0.93 |

**t_R:_** retention time; *m/z* (Expected): theoretical mass-to-charge ratio calculated from the molecular formula; *m/z* (Apex): experimentally observed value at the chromatographic peak apex; Δmass (ppm): mass error between theoretical and observed *m/z* values; N.d.: Not detected.
