# Supplementary material for: Comparative Phytochemical Profiling and Wound Healing Potential of Scabiosa pseudograminifolia Hub.‐Mor. and Scabiosa hololeuca Bornm.: UHPLC‐HRMS/MS Analysis and Fibroblast‐Based Evaluation
Source: Food Sci Nutr. 2026 Apr 8;14(4):e71738. doi: 10.1002/fsn3.71738 (PMC13058435; doi:10.1002/fsn3.71738)
Supplement: Supplementary file 3 — Data S3: Quantified Phenolic Compounds in the Methanol Extracts of the Aerial Parts of S. hololeuca. [file FSN3-14-e71738-s003.docx]

**Supplementary Material 3.** Quantified Phenolic Compounds in the Methanol Extracts of the Aerial Parts of S. hololeuca

| **Compound** | **t_R_ (min)** | **Moleculer formula** | **Exact mass**  **(M −H )^−^** | ***m/z* (Expected)** | ***m/z* (Apex)** | **Δmass (ppm)** | **MS/MS fragments** | $\boldsymbol{\mu}$**g_compound_/g_plant_**  _(medium_ _±std)_ |
| --- | --- | --- | --- | --- | --- | --- | --- | --- |
| 4-Hydroxybenzoic acid | 3.33 | C_7_H_6_O_3_ | 137.0244 | 137.02442 | 137.02434 | 0.60012 | 93.03465; 137.02458 | 123.46±9.40 |
| 4-O-Caffeoylquinic acid | 4.04 | C_16_H_18_O_9_ | 353.0878 | 353.08781 | 353.08777 | 0.11738 | 93.03465; 135.04536; 173.04573; 179.03552; 191.05641 | 2891.07±117.13 |
| Caffeic acid | 4.02 | C_9_H_8_O_4_ | 179.03498 | 179.03498 | 179.03496 | 0.12352 | 89.03974; 107.05019; 134.03746; 135.04532; 179.03508 | 832.43±0.92 |
| Gentisic acid | 3.26 | C_7_H_6_O_4_ | 263.1288 | 153.01933 | 153.01924 | 0.57945 | 179.0349; 161.0243; 135.0440; 133.0290; 107.0128; 89.0397; 71.0134 | 58.26±0.96 |
| Chlorogenic acid | 3.86 | C_16_H_18_O_9_ | 353.08781 | 353.08781 | 353.08774 | 0.20381 | 59.01391; 85.02959; 93.03465; 127.04025; 191.05641 | 14150.41±26.12 |
| Gallic Acid | 1.11 | C_7_H_6_O_5_ | 169.01425 | 169.01425 | 169.01419 | 0,35101 | 69.03465; 81.03461; 97.02975; 125.02446 | N.d. |
| *p*-Coumaric acid | 4.82 | C_9_H_8_O_3_ | 163.0395 | 163.04007 | 163.04002 | 0.28334 | 91.05555; 93.03455; 119.05032 | 97.16±2.24 |
| Protocatechuic acid | 2.2 | C_7_H_6_O_4_ | 153.0193 | 153.01933 | 153.01924 | 0.57945 | 81.03468; 91.01912; 108.02184; 109.0296 | 357.05±4.30 |
| Apigenin-7-O-Glc | 6.01 | C_21_H_20_O_10_ | 432.10555 | 431.09837 | 431.09821 | 0.38143 | 63.02412; 107.01403; 117.03482; 211.0407; 268.03809 | 421.14±4.38 |
| Apigenin | 7.31 | C_15_H_10_O_5_ | 270.05282 | 269.04555 | 269.04553 | 0,06606 | 65.0034; 107.01399; 117.03474;149.02464; 151.00383 | N.d. |
| Diosmetin | 7.39 | C_16_H_12_O_6_ | 299.0561 | 299.05611 | 299.05591 | 0.67478 | 63.02407; 65.00331; 107.01419; 151.00371; 284.0329 | 73.61±0.63 |
| Hyperoside | 5.64 | C_21_H_20_O_12_ | 464.0955 | 463.0882 | 463.08844 | 0.51813 | 227.03535; 243.03008; 255.02994; 271.02515; 300.02786 | N.d. |
| Luteolin | 6.9 | C_15_H_10_O_6_ | 286.0404 | 285.04046 | 285.04034 | 0.40612 | 65.00335; 107.01403; 133.02962; 151.00354; 175.04062 | 147.68±1.07 |
| Naringin | 5.81 | C_27_H_32_O_14_ | 579.1719 | 579.17193 | 579.17065 | 2.20263 | 119.0495; 151.0038; 271.0611; 313.0717 | 7.29±0.00 |
| Vanilin | 4.49 | C_8_H_8_O_3_ | 151.04006 | 151.04007 | 151.03998 | 0.60893 | 108.02186; 151.04049 | 100.25±14.26 |

**t_R:_** retention time; *m/z* (Expected): theoretical mass-to-charge ratio calculated from the molecular formula; *m/z* (Apex): experimentally observed value at the chromatographic peak apex; Δmass (ppm): mass error between theoretical and observed *m/z* values; N.d.: Not detected.
