# Supplementary material for: Comparative Phytochemical Profiling and Wound Healing Potential of Scabiosa pseudograminifolia Hub.‐Mor. and Scabiosa hololeuca Bornm.: UHPLC‐HRMS/MS Analysis and Fibroblast‐Based Evaluation
Source: Food Sci Nutr. 2026 Apr 8;14(4):e71738. doi: 10.1002/fsn3.71738 (PMC13058435; doi:10.1002/fsn3.71738)
Supplement: Supplementary file 4 — Data S4: Quantified Phenolic Compounds in the Aqueous Extracts of the Aerial Parts of S. hololeuca. [file FSN3-14-e71738-s001.docx]

**Supplementary Material 4.** Quantified Phenolic Compounds in the Aqueous Extracts of the Aerial Parts of S. hololeuca

| **Compound** | **t_R_ (min)** | **Molecular formula** | **Exact mass**  **(M −H )^−^** | ***m/z* (Expected)** | ***m/z* (Apex)** | **Δmass (ppm)** | **MS/MS fragments** | $\boldsymbol{\mu}$**g_compound_/g_plant_**  _(medium_ _±std)_ |
| --- | --- | --- | --- | --- | --- | --- | --- | --- |
| 4-Hydroxybenzoic acid | 3.31 | C_7_H_6_O_3_ | 137.0244 | 137.02442 | 137.02432 | 0.71148 | 65.03989; 93.03471; 137.0246 | 148.23±0.46 |
| 4-O-Caffeoylquinic acid | 4.04 | C_16_H_18_O_9_ | 353.0878 | 353.08781 | 353.08771 | 0.29024 | 93.03463; 135.04526; 173.04568; 179.03542; 191.05635 | 1906.38±34.83 |
| Abscisic acid | 6.58 | C_15_H_20_O_4_ | 263.1288 | 263.12888 | 263.12885 | 0.01322 | 122.0375; 153.09232; 203.10783; 204.11591; 219.13928 | N.d. |
| Caffeic acid | 4.05 | C_9_H_8_O_4_ | 179.03498 | 179.03498 | 179.03497 | 0.03829 | 89.03975; 107.05039; 134.03752; 135.04535; 179.03532 | 6384.86±22.80 |
| Gentisic acid | 3.24 | C_7_H_6_O_4_ | 153.0193 | 153.01933 | 153.01926 | 0.47973 | 109.0292; 81.0345 | 245.09±18.24 |
| Chlorogenic acid | 3.86 | C_16_H_18_O_9_ | 353.08781 | 353.08781 | 353.08765 | 0.46310 | 59.0139; 85.02959; 93.03463;127.04015; 191.05635 | 1307.87±29.77 |
| Gallic Acid | 1.11 | C_7_H_6_O_5_ | 169.01425 | 169.01425 | 169.01422 | 0.17045 | 69.03464; 79.01901; 81.03467; 97.02957; 125.02454 | N.d. |
| *p-*Coumaric acid | 4.81 | C_9_H_8_O_3_ | 163.0395 | 163.04007 | 163.04004 | 0.18975 | 65.03979; 91.05544; 93.03466; 104.02679; 119.05034 | 747.55±11.26 |
| Protocatechuic acid | 2.2 | C_7_H_6_O_4_ | 153.0193 | 153.01933 | 153.01927 | 0.38001 | 65.00333; 81.03466; 91.01907; 108.02187; 109.02962 | 1640.79±23.92 |
| Quinic acid | 0.52 | C_7_H_12_O_6_ | 191.05611 | 191.05611 | 191.05609 | 0.09783 | 85.02964; 93.03469; 109.02962; 127.04018; 173.04582 | N.d. |
| Apigenin-7-O-Glc | 5.99 | C_21_H_20_O_10_ | 432.10555 | 431.09837 | 431.09827 | 0.23985 | 63.02413; 107.01399; 117.03472; 211.04036; 268.03809 | N.d. |
| Diosmetin | 7.39 | C_16_H_12_O_6_ | 299.0561 | 299.05611 | 299.05582 | 0.98092 | 107.01398; 151.0036; 284.03305 | 84.89±0.94 |
| Hyperoside | 5.66 | C_21_H_20_O_12_ | 464.0955 | 463.1383 | 463.08859 | 0.84763 | 227.03568; 243.03008; 255.03023; 271.02515; 300.02789 | N.d. |
| Luteolin | 6.9 | C_15_H_10_O_6_ | 286.0404 | 285.04046 | 285.04034 | 0.40612 | 65.0034; 107.01404; 133.02969; 151.00388; 175.04036 | 150.99±4.47 |
| Vanilin | 4.49 | C_8_H_8_O_3_ | 151.04006 | 151.04007 | 151.04001 | 0.40688 | 91.0184; 107.0128; 108.0210; 123.0444; 136.0160 | 92.15±2.09 |

**t_R:_** retention time; *m/z* (Expected): theoretical mass-to-charge ratio calculated from the molecular formula; *m/z* (Apex): experimentally observed value at the chromatographic peak apex; Δmass (ppm): mass error between theoretical and observed *m/z* values; N.d.: Not detected.
